# Healthy life expectancy by county, race, and ethnicity in the USA, 2009–19: a systematic analysis of health disparities

**DOI:** 10.1016/j.lana.2025.101064

**Published:** 2025-04-17

**Authors:** GBD US Health Disparities Collaborators, Chris A. Schmidt, Amal A. Abdi, Farah Mouhanna, Ethan Kahn, Kelly Compton, Mathew M. Baumann, Yekaterina O. Kelly, Parkes Kendrick, Dillon O. Sylte, Zhuochen Li, Horacio Chacon-Torrico, Wichada La Motte-Kerr, Farah Daoud, Theo Vos, Simon I. Hay, Paula D. Strassle, George A. Mensah, Elizabeth Arias, David M. Murray, Frank C. Bandiera, Stephanie M. George, Eliseo J. Pérez-Stable, Christopher J.L. Murray, Ali H. Mokdad, Laura Dwyer-Lindgren

**Keywords:** Healthy life expectancy, HALE, USA, Health disparities, Racial and ethnic health disparities, Place based health disparities, BRFSS

## Abstract

**Background:**

There are substantial disparities in longevity in the USA; however, differences in healthy life expectancy (HALE) remain incompletely understood. We aimed to examine trends and disparities in HALE by race and ethnicity for 3 110 US counties.

**Methods:**

We used data from the American Community Survey (2009–19, N = 23.9 m–30.5 m, across indicators), Behavioral Risk Factor Surveillance System (2011–19, N = 1.7 m–3.9 m), Gallup Daily Survey (2009–16, N = 1.8 m–1.9 m); sociodemographic covariates; and disease-specific mortality rates in small-area estimation models to estimate Years Lived with Disability (YLDs) by county, race and ethnicity (American Indian or Alaska Native [AIAN], Asian or Pacific Islander [Asian], Black, Latino or Hispanic [Latino], and White), sex, age, and year (2009–19). We derived HALE using these YLD rates to discount life expectancy.

**Findings:**

From 2009 to 2019, HALE declined nationally by 0.3 years (95% uncertainty interval: 0.1–0.5) to 66.2 (62.7–69.4), while life expectancy increased by 0.5 years (0.5–0.5) to 79.1 (79.1–79.1). The Asian population had the highest HALE (72.3 [68.5–75.5] in 2019), followed by the Latino (68.5 [64.7–71.8]), White (65.9 [62.3–69.1]), Black (63.4 [60.1–66.3]), and AIAN populations (60.7 [57.0–64.1]). The Latino population had the longest absolute duration in poor health (13.8 years [10.4–17.6] in 2019) whereas the AIAN population had the longest proportion of life in poor health (17.3% [13.0–22.2]). From 2009 to 2019, HALE declined for the AIAN (by 1.4 years [1.0–1.8]) and White (by 0.6 years [0.4–0.8]) populations and the proportion of life in poor health increased for all populations. At the county level, HALE declined in 91.3% of counties (2 812 of 3 079; 65.6% statistically significant) from 2000 to 2019 and ranged from 55.1 to 76.2 years in 2019. Racial and ethnic disparities at the county level were broadly similar to national patterns, but with some exceptions.

**Interpretation:**

Disparities in HALE in the USA are large, and HALE has worsened for many populations in recent years. The expanding duration and proportion of life spent in poor health could indicate an increasing need for health services.

**Funding:**

Intramural Research Program, 10.13039/100006545National Institute on Minority Health and Health Disparities, US National Institutes of Health (contract #75N94023C00004). Intramural Research Program, National Institute on Minority Health and Health Disparities; 10.13039/100000050National Heart, Lung, and Blood Institute; Intramural Research Program, National Cancer Institute; 10.13039/100000049National Institute on Aging; 10.13039/100000069National Institute of Arthritis and Musculoskeletal and Skin Diseases; 10.13039/100006085Office of Disease Prevention; and 10.13039/100000118Office of Behavioral and Social Sciences Research, US National Institutes of Health (contract #75N94019C00016).

**Research in context**

**Evidence before this study**

Pronounced disparities have previously been identified in life expectancy, disease prevalence, and self-rated health in the United States (USA) by race, ethnicity, and location. For example, a recent study documented racial disparities across most counties in the USA, with Asian and Latino populations typically having relatively high life expectancy and AIAN and Black populations typically having relatively low life expectancy, but with considerable variation among counties. Healthy life expectancy (HALE), which accounts for both mortality and morbidity in a population, has been described in the USA and varies considerably among states and by race and ethnicity. We searched PubMed from inception to March 4, 2025, using the search string (“healthy life expectancy” [Title/Abstract] OR “health adjusted life expectancy” [Title/Abstract]) AND (United States [Title/Abstract] OR US [Title/Abstract] OR U.S. [Title/Abstract]) AND (race [Title/Abstract] OR ethnicity [Title/Abstract] OR American Indian [Title/Abstract] OR Alaska Native [Title/Abstract] OR Asian [Title/Abstract] OR Black [Title/Abstract] OR Hispanic [Title/Abstract] OR Latino [Title/Abstract] OR Pacific Islander [Title/Abstract] OR White [Title/Abstract] OR subnational [Title/Abstract] OR region [Title/Abstract] OR geographic [Title/Abstract] OR state [Title/Abstract] OR county [Title/Abstract]) for studies examining geographical or racial and ethnic disparities in HALE in the US. Several studies documented considerable geographical disparities in HALE at the census region or state level; however, no studies comprehensively examined HALE at the county level. Similarly, multiple studies have found substantial racial and ethnic disparities in HALE; however, most focus on White, Latino, and Black populations only. Only one study—at the census region (N = 4) level—examined geographical and racial and ethnic disparities in HALE simultaneously.

**Added value of this study**

We estimated HALE by county for five mutually exclusive racial and ethnic populations (American Indian or Alaska Native [AIAN], Asian or Pacific Islander [Asian], Black, Latino or Hispanic of any race [Latino], and White) in the USA from 2009 to 2019. This study is the first to estimate HALE over multiple years at the county level and to provide estimates for the AIAN and Asian populations alongside Black, Latino, and White populations, allowing us to examine trends over time in years lived in good versus poor health at a previously unavailable spatial, temporal, and demographic resolution. This study is also the first to incorporate a broad suite of health indicators to predict HALE in the USA by race and ethnicity. These results are made available for further research and provide a critical springboard for further detailed examinations of health disparities.

**Implications of all the available evidence**

There are large geographical and racial and ethnic disparities in HALE in the USA. While the trends and disparities in HALE are similar to those observed for life expectancy, they differ in key ways, underscoring the importance of considering health status in addition to longevity. Decreases in HALE in some racial and ethnic populations and in many counties, as well as increases in the proportion of life spent in poor health across all racial and ethnic populations point to a growing burden of morbidity. This in turn may indicate an expanding need for the types and quantity of health care services, especially considering the continued growth and aging of the US population. These results highlight the complex interplay between mortality and morbidity and may inform efforts to address disparities in the USA and their causes.

## Introduction

Health disparities by race, ethnicity, and location have been documented in the United States,^1^, ^2^, ^3^, ^4^, ^5^ providing guidance for possible interventions to support populations at greatest risk for reduced longevity or poor health. However, population health is often viewed through a narrow lens (eg, a single disease) that may not reflect the totality of health experiences over the life course. Healthy life expectancy (HALE) is a summary measure of population health that incorporates information about mortality and morbidity at all ages.^6^ HALE is expressed in years, analogous to life expectancy, and represents the average amount of time lived in good health. HALE is estimated by discounting each year of life by the expected degree of health loss during that year from disease or injury, providing a metric equal to life expectancy minus years lived in poor health. HALE, years lived in poor health, and life expectancy together reveal the extent to which loss of healthy life is driven by premature mortality versus ill health. Comparing these metrics across populations can provide a more complete picture of health disparities and ensure that public health policies are informed by a comprehensive view of unique population health circumstances.

Previous analyses of health disparities in the United States have reported substantial geographic disparities (eg, among US counties) and racial and ethnic disparities in life expectancy,^3^ cause-specific mortality,^5^ and the prevalence of some diseases and risk factors.^7^, ^8^, ^9^, ^10^ Relatively few studies to date have quantified HALE in the USA. Existing estimates have been reported at the national or state level for the total population^11^, ^12^, ^13^; or for subsets of years, ages, races and ethnicities, or specific forms of disability (eg, cognitively healthy life expectancy^14^), sometimes using only a single indicator of health to estimate non-fatal burdens.^15^, ^16^, ^17^, ^18^, ^19^ There remains a need for high-spatial-resolution estimates of HALE in the USA by race and ethnicity over multiple years while accounting for the diverse conditions contributing to poor health. Disparities in HALE are likely to be larger across counties, races and ethnicities considered together than by these dimensions alone, so analyzing HALE simultaneously by geography, race and ethnicity is an essential step toward identifying specific populations that are especially impacted by poor longevity and health.

Our study addresses this information gap by providing a comprehensive and detailed analysis of HALE in the USA. The health indicators employed in deriving HALE vary across the literature, but we mirror the methodological approach of the Global Burden of Diseases, Injuries, and Risk Factors Study (GBD) 2021,^12^ using Years Lived with Disability (YLDs) to discount life expectancy by cumulative health loss from hundreds of diseases and injuries, providing a more complete accounting of health than is possible from reliance on only a small number of generic health indicators. We estimated HALE and years lived in poor health annually from 2009 to 2019 by US county, race, and ethnicity. These estimates could support efforts to monitor the effectiveness of public health programs in different populations. The results may also inform interventions at a range of spatial scales by helping to reveal drivers of premature death, poor health, and associated disparities that continue to hinder the realisation of long, healthy lives for all people.^20^

## Methods

This study complies with the Guidelines for Accurate and Transparent Health Estimates Reporting (Supplementary Table S1).^21^ This research received institutional review board approval from the University of Washington. No primary data were collected for this study, and we had no contact with human subjects.

### Units of analysis and overview of approach

We estimated HALE by county, race and ethnicity, sex, and age annually from 2009 to 2019 (Supplementary Figure S1). Our analysis begins in 2009 due to data limitations in earlier years (Supplementary Methods, pp 3–4). We used the same units of analysis as previous studies of mortality and life expectancy by county, race, and ethnicity.^3^^,^^5^ Spatial units consisted of 3 110 counties or merged-county units with stable boundaries over time,^22^ which we refer to as “counties” (Supplementary Methods, pp 5–6; Supplementary Table S2). Racial and ethnic classification (“race and ethnicity”) included five mutually exclusive populations: American Indian or Alaska Native (AIAN), Asian or Pacific Islander (Asian), Black, Latino or Hispanic of any race (Latino), and White, consistent with the 1977 version of the Office of Management and Budget standards for federal data collection on race and ethnicity.^23^ Although a 1997 update^24^ to these standards separated the Asian and Native Hawaiian or Pacific Islander (NHPI) populations and required the option for respondents to report multiple races, we used the simpler classification due to constraints in the mortality data used in prior analyses whose results are integral inputs to the present study.^3^^,^^5^ While we combined Asian and NHPI populations into one category, our estimates are more reflective of the health status of Asian individuals than NHPI individuals because the Asian population in the USA is an order of magnitude larger.^25^ We did not model health outcomes for a “Two or More Races” category but redistributed this population to single racial and ethnic populations using published models that bridge to a “primary race” for each individual reporting multiple races (Supplementary Methods, pp 3–4).^26^^,^^27^

### Health and sociodemographic indicators: deriving predictors of non-fatal burden

To provide predictors of morbidity, we obtained prevalence data for 15 disease-specific or generic self-reported health indicators (eg, lifetime asthma or frequent activity limitation) from the Behavioral Risk Factor Surveillance System (BRFSS; 2011–19),^28^ Gallup Daily (2009–16),^29^ and American Community Survey (ACS; 2009–19),^30^ chosen based on data availability, hypothesised relationships to morbidity, and lack of strong collinearity with other indicators (Supplementary Tables S3–S4). Sample size varied among indicators derived from a given survey source, depending on missingness, survey coverage, and data exclusions: ACS (N = 23.9 m–30.5 m, across indicators), BRFSS (N = 1.7 m–3.9 m), and Gallup (N = 1.8 m–1.9 m). We fit separate small-area models for each indicator to produce prevalence estimates for the full set of counties, years, and demographic strata targeted by this study (Supplementary Methods, pp 10–19; Supplementary Table S5; Supplementary Figure S2). These models were hierarchical generalized linear models with autocorrelated random effects to account for spatial correlation, as well as correlation across years, ages, and educational categories. Models were fit using the Template Model Builder package^31^ in R version 3.6.1. For data reported at higher aggregation levels than our modelling targets, we used a regression approach^32^ that treats outcomes in aggregate strata as population-weighted averages of constituent strata (Supplementary Methods, pp 15–17). Using data from the US decennial census,^25^ ACS, and the National Center for Health Statistics, we also derived several county-level sociodemographic covariates: (1) total population density; (2) proportions of birth outside the USA; (3) poverty; (4) income per capita; (5) high school education or higher; and (6) college education (BA or higher)—all but population density available by both county and race and ethnicity, but requiring small-area models to estimate for all strata (Supplementary Methods, pp 6–9; Supplementary Table S6).

### Years lived with disability rates: quantifying poor health

Next, we used the health and sociodemographic indicators as predictors of population-level disability rates. GBD has employed the concept of YLDs—a metric which combines the prevalence, duration, and severity of a cause (disease or condition)—to quantify the magnitude and duration of resulting disability (or synonymously for the present study, “poor health”) experienced by a given population, where disability is broadly defined to mean anything less than full mental and physical well-being and functioning. In the GBD framework, causes are assigned “disability weights” from 0 (no negative impact) to 1 (equivalent to death), indicating proportional loss of health.^12^ For example, severe low back pain with leg pain has a disability weight of 0.325, reflecting substantial health impacts; mild upper respiratory infections have a much lower disability weight of 0.006.

To estimate cause-specific YLD rates by county and racial and ethnic population, we fit models to GBD YLD estimates^12^ by age, sex, year, and state (the highest resolution available) for 16 groups of conditions which collectively comprise all causes of morbidity in GBD 2021 (Supplementary Methods, p 19; Supplementary Table S7). These groups represent large categories of related conditions—eg, neoplasms or mental disorders—chosen to provide a comprehensive but manageable set of modelling targets. To leverage correlations between mortality and morbidity, we included cause-specific mortality estimates (Years of Life Lost [YLL] rates) from a previous study^5^ as covariates. We regressed YLD rates from the GBD study on the county, race and ethnicity, sex, age, and year-specific YLL rates, health indicators, and sociodemographic indicators, using the same regression approach described above. Uncertainty in the input and outcome variables was propagated by fitting 50 models per cause, each using one draw (simulation) from the approximated posterior distribution of each modelled indicator and outcome. These draws were then aggregated from the 50 models to yield 1000 total draws per cause (Supplementary Methods, pp 19–23; Supplementary Figure S3).

We next summed YLD rates across the 16 causes to derive 1000 draws of all-cause YLD rates. To ensure consistency with GBD, we scaled these model estimates such that the population-weighted average of the county and race and ethnicity-level estimates matched the corresponding state-level estimates from GBD 2021 for each sex, age, year, and draw.

### Healthy life expectancy: calculating expected years of full health equivalent

Finally, we calculated HALE by draw and stratum (county, race and ethnicity, age, sex, and year) using Sullivan's method,^6^ as implemented by GBD (Supplementary Methods, pp 23–24).^12^ This process involved adjusting life table estimates (available from a previous study^5^ at the same stratification as our YLD estimates) by our scaled all-cause YLD rate estimates to produce 1000 draws of HALE by county, race and ethnicity, sex, age, and year. We used population weights to aggregate estimates for males and females to obtain estimates for all sexes combined, and similarly aggregated county-level estimates to the national level. We calculated final point estimates from the draw means, with 95% uncertainty intervals (UIs) derived from the 2.5th and 97.5th percentiles. We describe differences between populations and changes over time as statistically significant when the posterior probability that the difference is greater than zero is less than 2.5% or greater than 97.5%, analogous to a two-tailed test with an alpha of 0.05. Estimates are correlated over time and among racial and ethnic populations, such that differences can be statistically significant even when the uncertainty intervals for HALE in the two time periods or populations overlap substantially. We calculated years lived in poor health as the difference between life expectancy and HALE, and proportion of life in poor health as years lived in poor health divided by life expectancy. We estimated HALE by age and sex, but focus our reporting on HALE at birth (“HALE”) for all sexes combined. While we report some general geographic patterns that are apparent in our results, we did not conduct formal clustering analyses.

We mask modelled estimates for any county and racial and ethnic population with a mean annual population of fewer than 1000 from 2000 to 2019, as the mortality estimates in our HALE calculation were less reliable below this threshold per prior validation analyses (Supplementary Table S8).^3^

### Role of the funding source

Co-authors employed by the US National Institutes of Health contributed to data interpretation and revising drafts of this report. Otherwise, the funders had no role in study design, data collection, data analysis, or the initial writing of the report.

## Results

All differences reported here are statistically significant unless otherwise noted. For brevity, we focus on results for HALE at birth for all sexes combined; however, results by sex and age for all counties, years, and racial and ethnic populations are available from the Global Health Data Exchange (GHDx) website (https://ghdx.healthdata.org/record/ihme-data/us-hale-county-race-ethnicity-2009-2019). This study covers the entire US resident population, which grew from 306 million to 330 million from 2009 to 2019. Summed across all years in the study period, the study population was 50.8% female and 49.2% male; and 0.8% AIAN, 5.8% Asian, 13.0% Black, 17.3% Latino, and 63.1% White.

### National racial and ethnic disparities in HALE

**Fig. 1:**
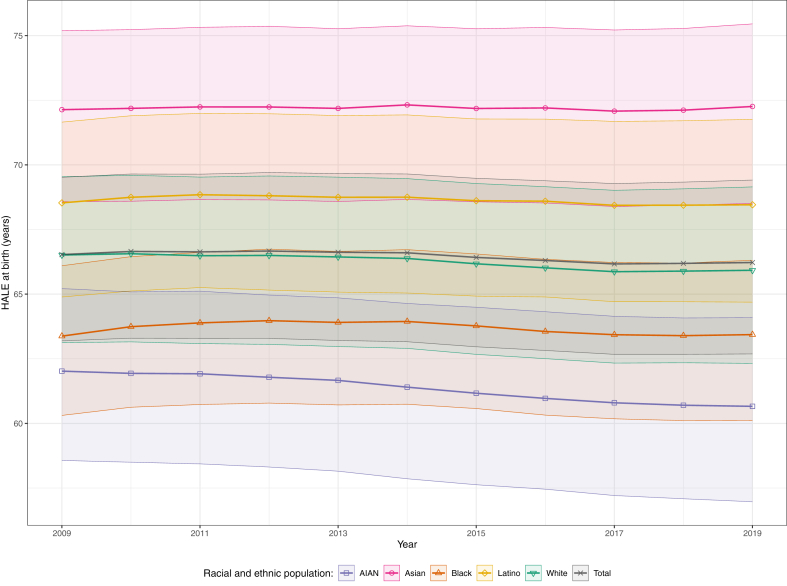
**Estimated healthy life expectancy in the USA, 2009–19, by year and racial and ethnic population**. Shaded areas with solid borders indicate 95% uncertainty intervals.

Between 2009 and 2019, HALE for the total US population decreased by 0.3 years (95% UI 0.1–0.5), from 66.5 (63.2–69.5) to 66.2 (62.7–69.4) years (Fig. 1). In all years, the Asian population had the highest HALE, followed by the Latino, White, Black, and AIAN populations, but with variable trends; differences between all racial and ethnic groups were statistically significant in all years. From 2009 to 2019, HALE decreased for the AIAN (by 1.4 years [1.0–1.8], from 62.0 years [58.6–65.2] to 60.7 years [57.0–64.1]) and White (by 0.6 years [0.4–0.8], from 66.5 [63.1–69.5] to 65.9 [62.3–69.1]) populations, but shifts were small and not statistically significant for the Asian (+0.1 years [−0.1 to 0.4], from 72.1 [68.6–75.2] to 72.3 [68.5–75.5]), Latino (−0.1 years [−0.3 to 0.1], from 68.5 [64.9–71.7] to 68.5 [64.7–71.8]), or Black (+0.1 years [−0.2 to 0.3], from 63.4 [60.3–66.1] to 63.4 [60.1–66.3]) populations. These temporal trends led to widening gaps relative to the total population for all but the Black population: in 2019, HALE was higher for the Asian population than the total population by 6.0 years (5.5–6.5; an increase from 5.6 years [5.1–6.0] in 2009) and in the Latino population by 2.2 years (1.9–2.6; versus 2.0 years [1.6–2.4] higher in 2009), whereas HALE in 2019 was lower for the White population by 0.3 years (0.2–0.4; versus a statistically non-significant difference of 0.0 years [−0.1 to 0.1] in 2009), for the Black population by 2.8 years (2.5–3.1; an improvement from a 3.2 year [2.8–3.4] gap), and for the AIAN population by 5.6 years (4.4–6.8; widening from a 4.5 year [3.4–5.7] gap). HALE disparities decreased between the Black and Latino populations (narrowed from 5.2 years [4.5–5.7] to 5.0 years [4.4–5.6]), and Black and White populations (narrowed from 3.1 years [2.8–3.5] to 2.5 years [2.1–2.9]), but increased between all other racial and ethnic populations. These changes were statistically significant in all cases except a non-significant increase between the Black and Asian populations (disparity increased by 0.1 years [−0.3 to 0.1]). Thus, at the end of the study period there was an 11.6 year (10.3–13.0) difference between the racial and ethnic populations with the highest and lowest HALE (Asian and AIAN, respectively), an increase from the 10.1 year (8.9–11.4) difference in 2009.

**Fig. 2:**
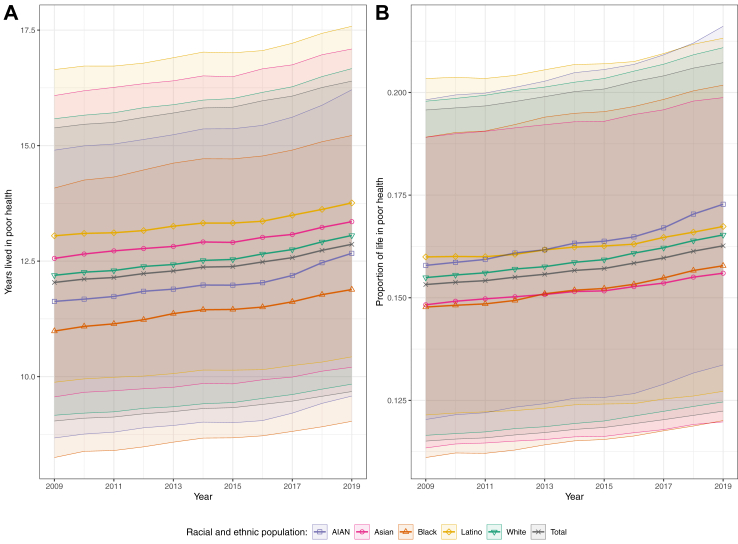
**Estimated (A) years lived in poor health and (B) proportion of life expectancy in poor health from birth in the USA, 2009–19, by year and racial and ethnic population**. Shaded areas with solid borders indicate 95% uncertainty intervals.

Years lived in poor health increased from 2009 to 2019 for the total population by 0.8 years (0.6–1.0), from 12.0 (9.0–15.4) to 12.9 years (9.7–16.4), while the proportion of life in poor health increased by 0.9 percentage points (0.7–1.2), from 15.3% (11.5–19.6) in 2009 to 16.3% (12.2–20.7) in 2019 (Fig. 2). These changes correspond with a shift in average US life expectancy from 78.6 years (78.5–78.6) in 2009—including 66.5 healthy years and 12.0 years in poor health—to 79.1 (79.1–79.1) years in 2019, with just 66.2 of those years in full health and 12.9 in poor health. Years lived in poor health similarly increased over this period for each racial and ethnic population, with increases ranging from 0.7 years (0.5–0.9) in the Latino population to 1.0 years (0.7–1.4) in the AIAN population. In contrast with HALE, the Latino population had the most years lived in poor health in all years (13.0 years [9.9–16.6] in 2009 and 13.8 years [10.4–17.6] in 2019; not statistically significantly higher than the Asian population in 2016), followed by the Asian (12.6 years [9.6–16.1] and 13.4 years [10.2–17.1]), White (12.2 years [9.2–15.6] and 13.1 years [9.8–16.7]), AIAN (11.6 years [8.7–14.9] and 12.7 years [9.6–16.2]), and Black (11.0 years [8.2–14.1] and 11.9 years [9.0–15.2]) populations. Differences in years lived in poor health between racial and ethnic populations were statistically significant except between the following populations in the indicated years: AIAN and Asian (2019), AIAN and Black (2014–2017), AIAN and White (2018–2019), Asian and Latino (2016), and Asian and White (2009, 2015–2019). The AIAN population had the highest proportion of life in poor health in 2019 (17.3% [13.0–22.2], increased from 15.8% [11.7–20.3] in 2009 and surpassing the Latino population in 2014, although differences between these populations were not statistically significant prior to 2019), followed by the Latino population (16.7% [12.6–21.4] versus 16.0% [12.1–20.5] in 2009), the White population (16.5 [12.4–21.1], up from 15.5% [11.6–19.8] in 2009), the Black population (15.8% [11.9–20.2] versus 14.8% [11.1–19.0] in 2009), and the Asian population (15.6% [11.9–20.0], increased from 14.8% [11.3–19.0] in 2009 and surpassed by the Black population in 2014, although differences between these populations were not statistically significant in any year). Differences in proportions of life in poor health between racial and ethnic populations were statistically significant except between the following populations in the indicated years: AIAN and Latino (2009–2018), AIAN and White (2009–2013, 2015–2016), Asian and Black (2009–2019), and Latino and White (2011–2012, 2014–2019).

### Geographic and racial and ethnic disparities in HALE at the county level

**Fig. 3:**
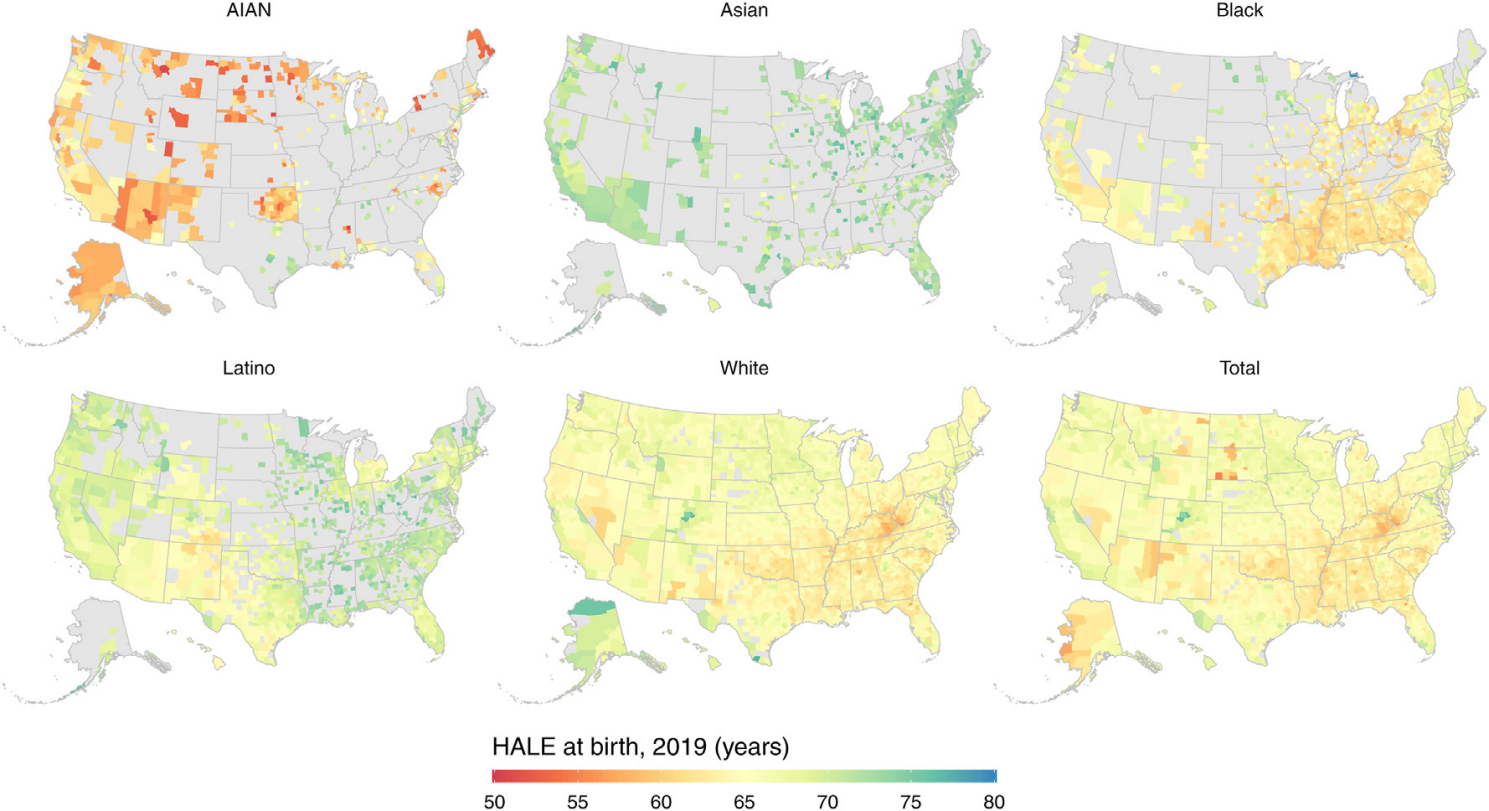
**Estimated healthy life expectancy estimates in 2019 by county and racial and ethnic population**. Estimates have been masked for county and racial and ethnic populations with a mean annual population fewer than 1000 people because mortality model performance declined notably below this threshold.

Across counties with unmasked estimates, HALE spanned a range of 21.2 years in 2019 for the total population, with the lowest county-level HALE at 55.1 years and the highest at 76.2 years (median 64.8 [interquartile range (IQR) 63.2–66.3]) (Fig. 3). The range of county-level HALE estimates was smaller than for the total population for the Asian (range of 12.4 years [from 64.9 to 77.3 years]; median 72.1 [IQR 70.8–73.4]) and Latino (range 16.7 years [60.3–77.0]; median 69.6 [IQR 67.5–71.4]) populations, and higher for the White (range 22.3 years [55.0–77.3]; median 64.8 [IQR 63.3–66.3]), Black (range 22.7 years [56.4–79.1]; median 62.7 [IQR 61.4–64.7]), and AIAN (range 24.5 years [50.1–74.6]; median 60.9 [IQR 58.0–64.8]) populations. Notably, the ten counties with the lowest mean HALE estimates for the AIAN population also had the ten lowest HALE estimates for any combination of county and racial and ethnic population, with the AIAN population in Neshoba County, Mississippi, having the lowest estimated HALE (50.1 years [46.9–53.0]) in the USA in 2019.

County-level racial and ethnic HALE disparities in 2019 echoed national trends (in which HALE was highest for the Asian population, followed by Latino, White, Black and AIAN populations) but with some local and regional exceptions (Fig. 3). For example, the Latino population had statistically significantly higher HALE than the Asian population in 44 of 658 counties with unmasked estimates (6.7%), spread across the Midwest and South, including eight counties in Wisconsin (the most of any state). In contrast, the Latino population had lower HALE than the White population in 51 of 1 469 counties (3.5%), particularly in Colorado (22 counties), Texas (12), and New Mexico (8), from a combination of particularly low HALE for the Latino population in southern Colorado and northern New Mexico, and particularly high HALE for the White population in central Colorado and southern Texas. The Black population had higher HALE than the White population in 139 of 1 485 counties (9.4%), widely distributed across the country but with notable counts of counties in Florida (14 counties), Georgia (10), Minnesota (11), and Texas (10). Finally, the AIAN population had higher HALE than the Black population in 82 of 327 counties (25.1%), including 14 counties in Texas.

**Fig. 4:**
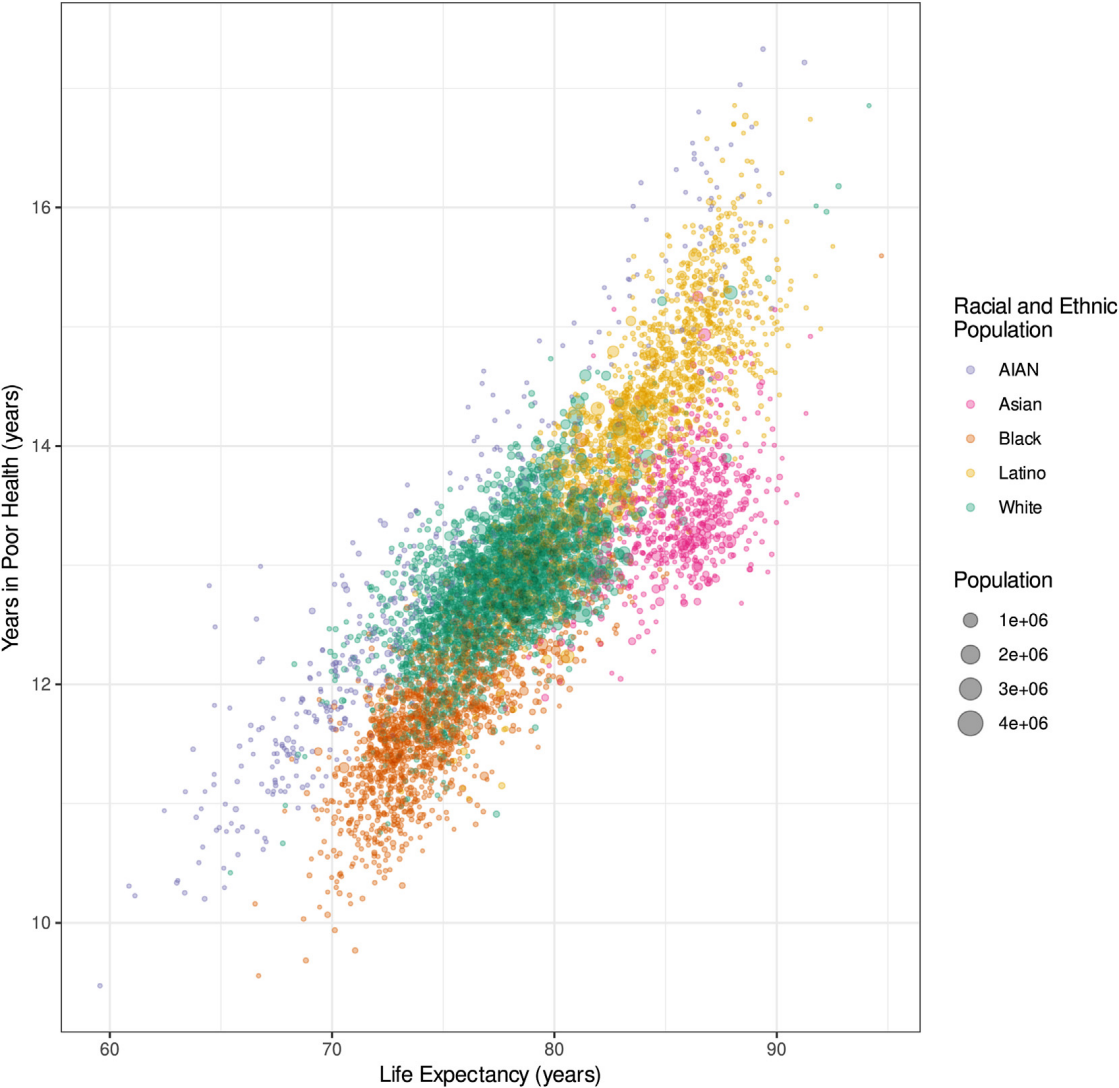
**Estimated county-level life expectancy versus years lived in poor health in 2019 by racial and ethnic population**. Estimates have been excluded for county and racial and ethnic populations with a mean annual population fewer than 1000 people because mortality model performance declined notably below this threshold.

Comparing life expectancy with years lived in poor health by county in 2019 revealed distinct patterns of morbidity and longevity by race and ethnicity, although with overlapping distributions (Fig. 4) which revealed pronounced disparities across counties even within single racial and ethnic populations. Life expectancies for the Asian and Latino populations tended to be higher than for the White population, and these were lower still for the Black and AIAN populations. At any given life expectancy, AIAN and Latino populations generally experienced more years in poor health than the Black and Asian populations, respectively. Conversely, for a given duration of years in poor health, the Asian and Black populations typically experienced several additional years of good health relative to the White and AIAN populations, respectively. These patterns reflect complex interactions on a county-level scale among race and ethnicity, geography, and both fatal and non-fatal health burdens.

**Fig. 5:**
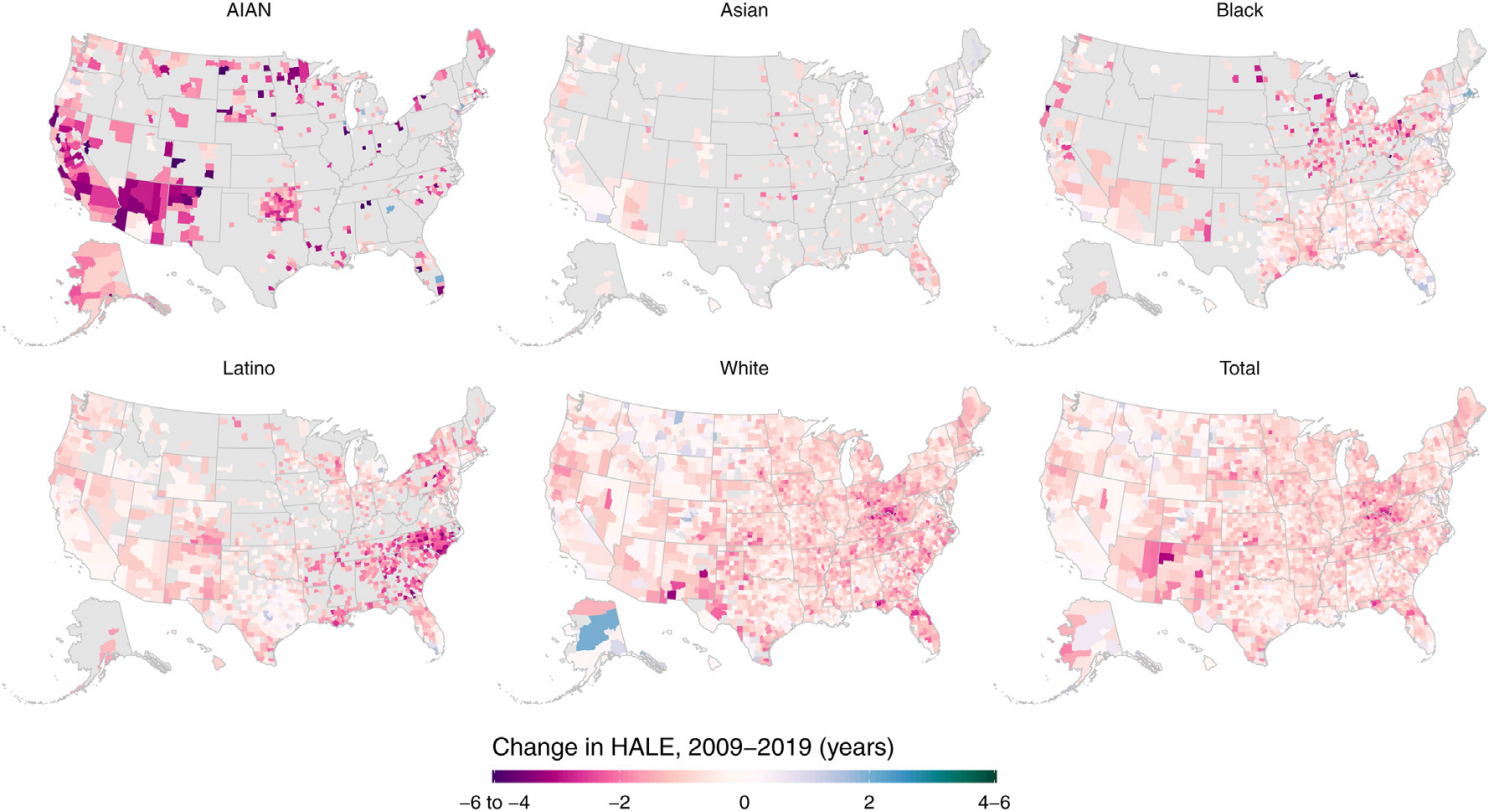
**Estimated change in healthy life expectancy from 2009 to 2019 by county and racial and ethnic population**. Estimates have been masked for county and racial and ethnic populations with a mean annual population fewer than 1000 people because mortality model performance declined notably below this threshold.

Most counties experienced statistically significant decreases in HALE from 2009 to 2019 for the total population (65.6% [2 019 of 3 079] of counties), with statistically significant increases in HALE in only 1.9% (58 counties; Fig. 5). In contrast, only 463 (15.0%) counties experienced a decline in life expectancy during this time, with 544 (17.7%) experiencing an increase. HALE had statistically significant declines from 2009 to 2019 in more counties for every racial and ethnic population than those in which HALE increased: HALE declined in 96 of 666 counties (14.4%) for the Asian population (versus 20 counties [3.0%] with increases, the remainder with no statistically significant changes); 558 of 1 478 counties (37.8%) for the Latino population (increased in 18 counties [1.2%]); 2 008 of 3 051 counties (65.8%) for the White population (increased in 52 counties [1.7%]); 412 of 1 487 counties (27.7%) for the Black population (increased in 45 counties [3.0%]); and 276 of 472 counties (58.5%) for the AIAN population (increased in 9 counties [1.9%]).

## Discussion

Our estimates of HALE and years lived in poor health suggest that the trend of declining HALE estimated for the United States by the GBD 2021 study^12^ was not uniformly distributed across US populations. Racial and ethnic disparities in HALE, years lived in poor health, and proportion of life in poor health were geographically widespread but of variable magnitude. HALE was substantially lower than life expectancy in every racial and ethnic population, indicating that poor health is a problem for all populations. However, the presence of disparities means that health loss from disease and injury is a larger problem for some populations than others. As a single measure combining the effects of mortality and morbidity in reducing healthy lifespans, HALE provides unique insights into the health experiences of each racial and ethnic population that are unavailable from life expectancy alone.

Alongside the estimated decreases in HALE, the USA concurrently experienced a trend of increasing disability rates, contrasting with a global trajectory toward stable or improving health status.^12^ The USA was the only high-income country to experience a decrease in HALE from 2009 to 2019 and had the ninth largest decrease in HALE during this period among all nations (of only 13 nations with a decrease in HALE).^12^ The GBD 2021 study ranked the USA 69th among 194 nations in terms of HALE in 2019,^12^ but the present analysis shows that many populations in the USA fare even worse in global comparisons. For example, in 2019 there were 149 (31.6%) counties for the AIAN population, 20 (1.3%) counties for the Black population, and 21 (0.7%) counties for the White population with lower HALE than three quarters of countries globally (25th percentile of country-level HALE estimates from GBD: 58.7 years).^12^

In this study, the AIAN population faced the greatest loss of healthy life by almost every measure. High morbidity has been noted for the AIAN population in previous studies of many conditions, including diabetes,^33^ obesity,^34^ and functional disability,^35^ and the AIAN population had among the highest mortality rates for most groups of causes in a recent study across all causes of death.^5^ From a broader sociohistorical perspective, AIAN communities have endured centuries of oppression, discrimination, and injustice.^36^ The resulting historical trauma has been tied to contemporary disparities, such as high rates of youth drug use and suicide.^37^ More generally, prior research has shown numerous pathways connecting racism and health, including socioeconomic status, residential segregation, chronic stress, mass incarceration, state-sanctioned violence, resource deprivation, and reduced health care and health insurance access.^38^, ^39^, ^40^, ^41^ These pathways likely contribute to the low HALE of the AIAN population, as well as the Black population. Our results underscore the profound disparities and worsening health affecting the AIAN population.

The Black population had lower HALE than the White population from 2009 to 2019, but this disparity narrowed due to a decline in HALE for the White population. The slowing gains in life expectancy for the White population over the past two decades have been attributed, in part, to increasing mortality rates due to overdoses and suicide (sometimes termed “deaths of despair”).^42^^,^^43^ Overdose deaths have also increased in the Black population, but the increase started later and has been slower, which may help explain the larger impact on HALE in the White population, and thus the reduced disparity between these populations.^5^^,^^44^, ^45^, ^46^ Previous studies have found indications of poorer health in the Black population than the White population, including worse general health^47^ and higher rates of functional disability,^35^ diabetes,^48^ and heart failure.^1^ On the other hand, higher prevalence has been noted in the White population for conditions such as chronic obstructive pulmonary disorder^49^ and low back pain.^50^ A recent study^51^ found that per capita spending on inpatient and emergency health services in 2016 in the USA was higher for Black individuals than for White individuals, suggesting delays in care-seeking by the Black population that are likely due in part to barriers in accessing such services—for example, high out-of-pocket costs due to high uninsured rates.

The Latino population—the largest minoritised racial and ethnic population in the country^52^—had the second-longest HALE nationally but among the highest proportions of life in poor health and the longest duration in poor health. Among Latinos, the combination of high life expectancy and high prevalence of chronic conditions such as obesity and diabetes in addition to low socioeconomic status has been examined extensively in the literature.^53^ Several possible explanations have been proposed, including differential migration by health status, reduced exposure to certain risk factors, and potentially protective psychosocial factors unique to this population.^54^, ^55^, ^56^ Evidence from previous studies on the relative levels of morbidity in the Latino population is mixed for a range of conditions (including diabetes, musculoskeletal disorders, and cardiovascular diseases), likely related to diversity within the Latino population with respect to birthplace, race, and socioeconomic status.^48^^,^^50^^,^^57^^,^^58^ Further research may help elucidate the combination of factors that may be driving both the higher longevity and proportion of life in poor health in the Latino population compared with many other racial and ethnic populations.

The Asian population had the highest HALE and one of the lowest proportions of life in poor health, consistent with prior research describing advantages in life expectancy and health for this population.^35^^,^^59^^,^^60^ The small size of the Asian population in much of the USA —and thus the high proportion of counties with masked estimates—makes broad geographical trends difficult to discern, but HALE estimates for the Asian population in Hawaiʻi are notably low. The NHPI population (included in the Asian population in this analysis) accounts for a relatively large proportion of the population in Hawaiʻi. Previous research has demonstrated poorer health outcomes and lower longevity in the NHPI population than the Asian population,^34^^,^^61^^,^^62^ including a study that estimated significantly lower HALE in Hawaiʻi for the Native Hawaiian population relative to the White population and certain Asian populations.^18^ The health disparities between Asian and NHPI populations noted by other studies underscore the importance of refining analyses of health disparities to avoid overshadowing the health needs of smaller populations like the NHPI population. While enjoying the longest and healthiest lives on average, the Asian population experienced among the highest durations lived in poor health, an expansion of morbidity that implies increased need for health care over the life course.

HALE estimates are sensitive to how disability or poor health are defined, and comparisons between studies need to be interpreted cautiously when approaches are inconsistent.^63^ With that caveat, our results complement previous estimates of HALE in the USA.^16^^,^^17^ Chang et al.^16^ reported higher HALE in 2008 in the White population than the Latino population, contrasting with our results for 2009 and later. This difference likely stems from reliance on self-rated general health in Chang et al. as the sole measure of health status. We excluded self-reported general health from our analysis because the Latino population reports worse general health than expected from more objective measures, possibly due to language-dependent differences in interpretation of survey response levels.^64^ Crimmins et al.^17^ similarly relied on self-reported frequent activity limitation to quantify disability burdens for the total population nationally from 1970 to 2010. That study did not derive estimates by racial and ethnic population, but its overall estimates of YLDs in 2010 are higher by a few years than the corresponding GBD 2021 estimates (and hence our study). This difference between our studies likely derives from their use of a single expansive binary health measure versus the detailed modelling of disease-specific burdens by GBD.^65^^,^^66^

This study has several important limitations. First, our HALE estimates have wide uncertainty, largely driven by uncertainty in GBD disability weights.^67^ However, since uncertainty is highly correlated across draws in our modelling process, comparisons among racial and ethnic populations, locations, and years often have statistical significance despite high uncertainty for individual strata. Second, no independent estimates exist for HALE by county and race and ethnicity, preventing external validation of our results. Third, some data sources reported coarser geographic, age, or race and ethnicity detail than what we modelled in this analysis. We designed our methodological approach specifically to leverage data regardless of resolution; however, it is likely that we have not fully recovered the variation present within the coarser strata used by these data sources. Fourth, we acknowledge that aspects of our approach, including the use of multiple data sources, bridged racial identification, modelled covariate estimates, and the use of modelled health indicators to estimate population-level disability rates, may introduce bias in our results. The extents of these biases and their impact on the results are difficult to quantify, but we have strived to incorporate the uncertainty inherent in these processes in our analytical methodology to the extent feasible. Fifth, the accuracy of the data inputs likely varies by race and ethnicity, which may differentially impact the accuracy of the results. For example, misclassification of race and ethnicity on death certificates is particularly impactful for the AIAN population^68^; while the life expectancy estimates used for this analysis correct for misclassification, it is possible some error remains. Sixth, we used a simplified classification of racial and ethnic populations due to data constraints, which likely masks important variation in HALE within these populations. Efforts to generate separate estimates for Asian, NHPI, and multiracial populations are ongoing. Seventh, our modelling approach assumes that the association between disability and a given predictor is constant across all racial and ethnic populations, locations, and years; violations of this assumption may lead to over or underestimation of differences or changes over time in HALE. Finally, our study covers only the pre-COVID period, but HALE in the USA was previously estimated to decline during the first two years of the pandemic by 1.8 years (2019–21).^13^ AIAN, Black, and Latino communities experienced disproportionate impacts from the COVID-19 pandemic,^69^^,^^70^ and we expect major disruptions to trends by race and ethnicity across all indicators. Nonetheless, our estimates through 2019 provide a valuable baseline with which to evaluate post-pandemic HALE, the varied impacts of the pandemic, and drivers of resulting disparities. We expect that the racial and ethnic differences and geographic patterns that we report through 2019 are a strong guide to current challenges regarding inequalities in healthy life expectancy.

Research on HALE has distinguished between scenarios of more, less, or stable time spent in poor health as life expectancies improve, with important implications for the allocation of health resources.^71^^,^^72^ The pairing of higher life expectancy for most racial and ethnic populations in the USA with more time spent in poor health mirrors the morbidity expansion identified for several other high-income countries in a recent review,^72^ and suggests an increasing demand for health care and supportive services, especially in the context of a growing and aging US population.^73^ Addressing these population-wide resource requirements to support good health and longevity for all, while simultaneously eliminating substantial disparities in HALE, is essential and will require a multipronged effort to identify and resolve both overt and insidious drivers of health injustice.

## Contributors

L Dwyer-Lindgren, A H Mokdad, and C J L Murray were responsible for the study concept and design. C Schmidt, A A Abdi, E Kahn, F Mouhanna, Y O Kelly, and M M Baumann extracted and processed the data inputs. C Schmidt, A A Abdi, and F Mouhanna wrote the computer code and designed and carried out the statistical analyses with input from L Dwyer-Lindgren. C Schmidt, Y O Kelly, and M M Baumann prepared the tables and figures. C Schmidt wrote the first draft of the paper. All authors contributed to writing subsequent versions of the manuscript, critically reviewed the methods and results, and approved the final version of the manuscript. L Dwyer-Lindgren, A H Mokdad, W La Motte-Kerr, and F Daoud managed the project. All authors provided intellectual inputs into aspects of this study. C Schmidt, A A Abdi, E Kahn, Y O Kelly, M M Baumann, and L Dwyer-Lindgren accessed and verified the data. L Dwyer-Lindgren, A H Mokdad, and C J L Murray had final responsibility for the decision to submit for publication.

## Data sharing statement

Estimates of HALE by county, race and ethnicity, sex, age, and year are available for download from the Global Health Data Exchange (https://ghdx.healthdata.org/record/ihme-data/us-hale-county-race-ethnicity-2009-2019). Information about the underlying data sources is available in the Supplementary Methods (pp 3–5) and Supplementary Tables S4–S6. The code used for this analysis is available on GitHub: https://github.com/ihmeuw/USHD.

## Editor note

The Lancet Group takes a neutral position with respect to territorial claims in published maps, figures, tables, and institutional affiliations.

## Declaration of interests

We declare no competing interests.
