# Supplementary material for: Healthy life expectancy by county, race, and ethnicity in the USA, 2009–19: a systematic analysis of health disparities
Source: Lancet Reg Health Am. 2025 Apr 17;45:101064. doi: 10.1016/j.lana.2025.101064 (PMC12137174; doi:10.1016/j.lana.2025.101064)
Supplement: Supplementary Methods, Figures and Tables [file mmc1.pdf]

Supplementary Appendix: Healthy life expectancy by county, race, and ethnicity in the USA, 2009–19: a systematic analysis of health disparities

Contents

Supplementary Methods ... 3

1. Data sources and processing ... 3
2. Spatial units ... 5
3. Covariate estimation and smoothing ... 6
4. Estimation of indicators of non-fatal burden ... 10

4.1 Model specification ... 10

4.2 Addressing data misalignment ... 15

4.3 Post-stratification frame ... 17

4.4 Hyperprior sensitivity analysis ... 18

5. YLD estimation ... 19

5.1 Cause hierarchy ... 19

5.2 Covariate selection ... 19

5.3 Model specification ... 20

5.4 Uncertainty propagation ... 22

5.5 Hyperprior sensitivity analysis ... 22

5.6 Calibration ... 23

6. HALE estimation ... 23

References ... 24

Supplementary Tables ... 27

Supplementary Table 1. GATHER Checklist ... 27

Supplementary Table 2. Counties combined to create historically stable units of analysis ... 29

Supplementary Table 3. Indicators of non-fatal burden ... 30

Supplementary Table 4. Health indicator survey data sources 33
Supplementary Table 5. Population data sources 38
Supplementary Table 6. Covariate data sources 39
Supplementary Table 7. Modelled causes of morbidity 42
Supplementary Table 8. Population mask........ 43
Supplementary Figures 44
Supplementary Figure 1. Data and modelling flowchart. 44
Supplementary Figure 2. Hyperprior sensitivity analysis (YLD indicators). 45
Supplementary Figure 3. Hyperprior sensitivity analysis (YLD models). 46

Supplementary Methods

The methods used for this analysis are an extension of methods previously developed for estimating all-cause and cause-specific mortality and life expectancy by county and racial and ethnic population in the USA.[1,2] A graphical overview of the analytical procedures is provided in Supplementary Figure 1.

1. Data sources and processing

Population data

We processed population data from various sources (Supplementary Table 5) to derive three sets of population estimates by county, age group (0, 1–4, 5–9, …, 80–84, 85+ years of age), sex (male or female), and year (2000–19): population separately by racial and ethnic population, educational attainment (four categories: Less than High School, High School, Some College, and BA Degree or Higher), and marital status (three categories: Currently Married, Formerly Married, and Never Married). Population totals by racial and ethnic population were derived from intercensal population estimates from Census data created by the National Center for Health Statistics (NCHS).[3,4] Population by educational attainment and population by marital status were derived from county-level population data from the US census and American Community Survey (ACS), stratified separately by age, sex, and educational attainment or marital status, plus state-level population distributions by marital status, educational attainment, age, sex, and year, from Public Use Microdata Series (PUMS) files.

We classified racial and ethnic populations in accordance with the standards for federal data collection on race and ethnicity issued in 1977 by the Office of Management and Budget (OMB).[5] Although these standards were updated in 1997 to require that federal data collection afford distinct Asian and Native Hawaiian or Pacific Islander (NHPI) populations, and the option to identify as multiple races, these changes were not fully realised on death certificates until 2018, and data on misclassification of racial and ethnic population on death certificates are only available using the 1977 OMB standards. It is also not possible to disaggregate Asian and NHPI populations on death certificates prior to 2011 due to the use of a combined Other Asian and Pacific Islander residual category. We therefore categorised racial and ethnic populations as American Indian or Alaska Native (AIAN), Asian or Pacific Islander (Asian), Black (Black), Latino or Hispanic of any race (Latino), and White (White). We used the imputed or “bridged” race estimates from NCHS, which derive from models[6] to predict “primary race” for individuals who report

multiple races, thereby bridging Census responses made using the 1997 race categories to the simpler 1977 categories that we used for our analysis.

Prevalence data

We extracted microdata (ie, unaggregated individual-level data) from the Behavioral Risk Factor Surveillance System (BRFSS),[7] Gallup Daily,[8] and American Community Survey (ACS).[9] These surveys employ complex designs to produce samples that are representative of the US population across all ages (ACS) or only adults (ages 18+, BRFSS and Gallup), either including institutionalised individuals (ACS, for the years considered in this analysis) or excluding them (BRFSS and Gallup). While ACS produces samples for the entire age range, some survey items are only relevant or available for a subset of ages. Single-year ACS microdata were obtained from IPUMS and represented partial samples of the full ACS survey. Gallup and ACS both provided full geographical coverage for the USA from 2009 to 2019, at the county (Gallup) or Public Use Microdata Area (PUMA; ACS) level, but data resolution for BRFSS was more variable, with county-level identifiers not uniformly provided in all states and years (Supplementary Tables 3–4). Due to major changes in survey design that prevented reliable construction of a full and consistent time series, we began our analysis in data year 2009, excluded Gallup data after survey year 2016 (the survey changed beginning in 2018, but the 2017 data for some variables showed substantial deviation from the preceding trend, and we were unable to ascertain whether this was artifactual), BRFSS data prior to 2011 (and for 2017 due to frequent heavy outliers in the data), and ACS data from 2010 (due to frequent outliers relative to other data years, possibly related to the decennial Census). We used the versions of racial and ethnic population coding variables provided by the survey series that conformed to the 1977 OMB guidelines, when possible, but otherwise combined separately reported Asian and NHPI groups. Gallup reported all race and ethnicity populations selected by individuals identifying as multiracial; we imputed a "primary race" for each of these individuals using bridging models similar to those described above for population data.[10]

We collapsed the survey microdata to produce stratum-level estimates for a suite of 15 self-reported health indicators (Supplementary Table 3), which include both generic indicators of health and disease-specific concepts. Some indicators were available from only a single survey, but several were available from both BRFSS and Gallup, with compatible question wording, and for these we extracted data from both surveys for simultaneous inclusion in the small-area estimation models (section 4). For each indicator and data source, we derived counts and sample sizes stratified by county, year, age group, sex, racial and ethnic population, educational attainment, and marital status. We produced survey-weighted

counts for each stratum to account for unequal sampling probability and nonresponse bias. We did not adjust uncertainty within these stratification categories because sample sizes were insufficient to consistently derive design effects at this high level of stratification.

YLD and mortality data

Estimates of Years Lived with Disability (YLDs) from the Global Burden of Diseases, Injuries, and Risk Factors Study (GBD) 2021[11] served as response variables in our small-area estimation models of YLD rates and as raking targets after model-fitting. GBD 2021 reports YLD estimates for the USA by state, year, age, and sex, for all races and ethnicities combined. We obtained 50 draws from the joint distribution of GBD YLD rates for 16 groups of conditions (referred to as "causes"), which collectively cover all causes of morbidity in the GBD 2021 study (section 5.1; Supplementary Table 7). We retrieved YLD rates separately for the major categories of non-communicable diseases and injuries, but we treated all communicable, maternal, and neonatal conditions as a single unit because of their comparatively small contribution to morbidity burden in the USA.

Mortality estimates served two functions in our analysis. First, rates of Years of Life Lost (YLL) were incorporated in small area estimation models as predictors of YLD rates. Separately, life tables were essential inputs in the final calculation of HALE. We obtained YLL and life table estimates by county, race and ethnicity, age, sex, and year, which were produced in previous work.[2] YLL rates were obtained for the same set of 16 causes for which we modelled YLD rates (excepting mental disorders, which do not have a fatal component in GBD), and were used individually in the YLD model for the same cause. In this way we used the associations between fatal and non-fatal burdens for a given cause to improve estimates of YLD rates.

2. Spatial units

Our analysis focused on county-level estimates of non-fatal burden and healthy life expectancy. However, a small number of counties underwent boundary changes during our model timeframe (or that of the mortality study upon which our analysis depended—ie 2000–19). We therefore combined a small number of counties into merged geographic entities with stable boundaries over time; we refer to these 3110 counties or merged-county units as "counties", collectively encompassing all 3143 original counties (the merged units are detailed in Supplementary Table 2).

ACS and BRFSS sometimes report microdata at a spatial resolution that is coarser than county level. IPUMS provides ACS microdata at the level of PUMAs, which are non-overlapping spatial units that only sometimes correspond with the boundaries of individual counties. To map these data to the county-level units that we modelled, we created synthetic spatial units by aggregating PUMAs until their collective boundaries exactly matched that of one or more counties. As the PUMA definitions used by ACS changed over time (in 2006 due to Hurricane Katrina, and in 2012 to reflect the 2010 Census), we performed this spatial aggregation process separately for each year from 2009 to 2019, incorporating the appropriate PUMA boundaries for a given year. This process resulted in 943 and 976 merged "PUMA-county" units for years 2009–11 and 2012–19, respectively.

BRFSS microdata were obtained through a combination of state-provided files and publicly available files, including Selected Metropolitan/Micropolitan Area Risk Trends (SMART) BRFSS. These sources differed in their spatial resolutions, variously identifying respondents by their state, county, Core-Based Statistical Area (CBSA) or metropolitan divisions, depending on the source, state, and year. We used the source that gave the highest geographical resolution for a given location and year. As we did for the ACS microdata, we developed aggregated geographic units for each model year to harmonise the spatial resolution of the available BRFSS data with the county-level spatial units that we were modelling. BRFSS microdata were then collapsed by the resultant "CBSA-county" units. In some state-years this process resulted in greater spatial detail in urban areas, with the bulk of rural areas falling into "state remainder" spatial units. We designed our modelling framework cognisant of this varied spatial resolution, using a regression approach that retains as much information as possible about spatial variation in health indicators (section 4.2).

3. Covariate estimation and smoothing

The small-area estimation models used in the present study leveraged observed relationships between modelled outcomes and sociodemographic and socioeconomic factors (covariates) to improve estimates for strata lacking robust outcome data. We used the following covariates stratified by county and race and ethnicity population: educational attainment (high school or higher, and bachelor's degree or higher), poverty rate, proportion born outside the US, and income per capita. We also used population density by county (Supplementary Table 6). The underlying data for the covariates stratified by both county and racial and ethnic population contained missing values and displayed instability and low precision for some strata with small populations. As covariate estimates are required for all location, year, and racial and ethnic population combinations to derive model predictions, small area models were developed to derive

smoothed covariate estimates in all years, counties, and racial and ethnic populations prior to including these covariates in the indicator and YLD models. Population density was incorporated in these models in its original (non-imputed) form due to its complete spatial and temporal coverage.

Covariate data were derived primarily from the American Community Survey (ACS) and decennial population census. Racial classifications in the tabulated ACS and census data included separate groups for Asian and for Native Hawaiian or Pacific Islander (NHPI) populations; covariate data for these populations were combined into a single population for consistency with the indicator and YLD models. Covariate data were also combined for merged counties to derive a stable location set matching that used in our final models (section 2; Supplementary Table 2). The ACS provides estimates of uncertainty as Margins of Error (MOE) at a confidence level of 90%, rather than providing variance estimates themselves. Per guidance from the Census Bureau,[12] we calculated variance as:

$$\mathrm{Var}(X_i) = \left(\frac{\mathrm{MOE}(X_i)}{1.645}\right)^2 \tag{1}$$

where X_i is the mean estimate for group i. Also following Census guidance, we calculated variances for merged geographic and demographic entities as the sum of the variances over component groups, assuming independence:

$$\mathrm{Var}(X_1 + X_2) = \mathrm{Var}(X_1) + \mathrm{Var}(X_2) \tag{2}$$

As the ACS and decennial census data were derived from population samples but are reported as values scaled to total population sizes, their effective sample sizes were estimated to appropriately scale sampling variance in the imputation models, using a three-pronged strategy. The long-form decennial census questionnaires in 1990 and 2000, from which the education, poverty, and born outside the US data were derived for those years, were collected from 20% and 17% population samples, respectively.[13] Effective sample sizes were therefore assumed to be 20% or 17% of the total population for each combination of county, year, and racial and ethnic population in 1990 and 2000, respectively.

Effective sample sizes for ACS data, for which variances were calculated as above, were estimated by the relationship between effective sample size, $\hat{n}_{eff}$, estimates of the population proportion, $\hat{p}$, and its variance, $\mathrm{Var}(\hat{p})$:[14]

$$\hat{n}_{eff} = \frac{\hat{p}(1-\hat{p})}{\mathrm{Var}(\hat{p})} \tag{3}$$

As observations with observed proportions of 0.0 or 1.0 yield effective sample sizes of 0.0, the reported population fractions ($\hat{p}$) for these county, year, and race and ethnicity combinations were transformed via an empirical logit transformation[15] and then inverse-transformed by use of the standard inverse logit function before calculating the corresponding effective sample sizes:

$$\hat{p}_{emp} = \mathrm{logit}^{-1}\left(\log\left(\frac{\hat{p}+\frac{\varepsilon}{2}}{1-\hat{p}+\frac{\varepsilon}{2}}\right)\right) \tag{4}$$

where logit^{-1} is the standard inverse logit function and ε is defined as the smallest non-zero proportion in the data set. Effective sample sizes were calculated by use of these transformed proportions and the original reported $\mathrm{Var}(\hat{p})$. Counts of individuals with the modelled outcome (attainment of a bachelor's degree or higher, living below the poverty line, or born outside the US) were then calculated for each row by multiplying their original reported proportions and estimated effective sample sizes.

Bayesian imputation models were fit in R-INLA[16] v.20.09.25 in R v3.5.1[17] with binomial likelihood models, and explicitly borrow strength over space, time, and racial and ethnic population:

$$Y_{j,t,r} \sim \mathrm{Binomial}\left(p_{j,t,r}, \hat{n}_{eff,j,t,r}\right) \tag{5}$$

$$\mathrm{logit}\left(p_{j,t,r}\right) = \beta_0 + \gamma_{1,j} + \gamma_{2,j,t,r} + \gamma_{3,j,t} + \gamma_{4,r} \tag{6}$$

where $Y_{j,t,r}$ is the estimated count of individuals in county *j*, year *t*, and racial and ethnic population *r* with the modelled outcome, among an effective sample size of $\hat{n}_{eff,j,t,r}$ with proportion $p_{i,t,r}$. Model terms consist of:

- β_0 is a global intercept;

- $\gamma_{1,j}$ is a random intercept for county with a Besag-York-Mollie-type prior (BYM2) combining a conditional autoregressive distribution for spatial autocorrelation, based on county adjacency, with independent-and-identically-distributed (IID) Gaussian distribution;
- $\gamma_{2,j,t,r}$ is a random intercept for county, racial and ethnic population, and year combinations with a first-order autoregressive (AR1) temporal prior, replicated by county and racial and ethnic population;
- $\gamma_{3,j,t}$ is a random intercept for county and year combinations with an AR1 temporal prior replicated by county;
- and $\gamma_{4,r}$ is a random intercept for racial and ethnic population with an IID Gaussian prior.

A relatively uninformative Normal(mean = 0.0, precision = 0.10) prior was used for the global intercept. Default INLA hyperpriors were used for the hyperparameters. The BYM2 parameterisation includes a parameter, φ, which indicates the contribution of the structured spatial effect to the marginal variance; $\mathbf{logit}(\varphi)$ had a penalised complexity (PC) prior[18] corresponding to $\Pr(\varphi < 0.5) = 0.5$, and the log precision of the BYM2 model had a PC prior corresponding to $\Pr(\sigma > 1.0) = 0.1$. The AR1 models had Gamma(shape = 1.0, inverse-scale = 5×10^{-5}) priors on the precision and Normal(mean = 0.0, precision = 0.15) priors on the logit of the 1-year lagged correlation (ρ). The race and ethnicity IID term had a Gamma(shape = 1.0, inverse-scale = 5×10^{-5}) prior on the precision. These default priors were used as we considered them suitably uninformative, in the absence of *a priori* information with which to establish more informative priors. In INLA, a Gaussian approximation strategy, an empirical Bayes integration strategy, and a step-length for hyperparameter gradient calculations of 1×10^{-3} were used for model fitting. Due to numerical instability in the model for the proportion born outside the US, a series of model fits were performed with iteratively decreasing values added to the diagonal of the joint precision matrix (100, 10, and 1, respectively) to derive starting parameter values for the final model run. Mean estimates from these covariate models were used as covariate estimates in the small area models for non-fatal burden indicators (section 4), while posterior draws from the covariate models were used as covariate estimates in the YLD models (section 5).

4. Estimation of indicators of non-fatal burden

4.1 Model specification

Small-area models were fit separately by sex for each survey-derived indicator of non-fatal burden (Supplementary Table 3). We estimated these indicators by educational attainment and marital status, in addition to the strata we report, to reduce bias due to survey non-response and to harmonise weighting schemes among ACS, BRFSS, and Gallup surveys, and over time within each data source. The following model specification was used for variables derived from a single data source (only one of ACS, Gallup or BRFSS):

$$Y_{j,t,a,r,e,m,w} \sim \text{Binomial}\left(p_{j,t,a,r,e,m,w}, n_{j,t,a,r,e,m,w}\right) \quad (7)$$

$$\text{logit}\left(p_{j,t,a,r,e,m,w}\right) = \boldsymbol{\beta}_0 + \boldsymbol{\beta}_1 \cdot \boldsymbol{X}_{1,j,t,r} + \sum_{t'=1}^{k_t} \sum_{a'=1}^{k_a} \left(\gamma_{1,j,t',a',r} \cdot S_{t'}(t) \cdot S_{a'}(a)\right) \quad (8)$$

$$+ \gamma_{2,j} + \gamma_{3,t,a,r,e,m|a\geq 20} + \gamma_{4,t,a,r} + \gamma_{5,r,e,m,w}$$

Priors:

$\gamma_1 \sim$ LCAR: LCAR: LCAR: IID$(\rho_{1,a'}, \rho_{1,t'}, \rho_{1,j}, \sigma_1)$

$\gamma_2 \sim$ LCAR(ρ_2, σ_2)

$\gamma_3 \sim$ LCAR: LCAR: IID: LCAR: IID$\left(\rho_{3,t}, \rho_{3,a}, \rho_{3,e}, \sigma_3\right)$

$\gamma_4 \sim$ LCAR: LCAR: IID$(\rho_{4,t}, \rho_{4,a}, \sigma_4)$

$\gamma_5 \sim$ IID: LCAR: IID: IID$(\rho_{5,e}, \sigma_5)$

Hyperpriors:

$\sigma^{-2} \sim \text{PC}(\sigma_0 = 5, \alpha = 0.05) \rightarrow \Pr(\sigma > 5) = 0.05$

$\text{logit}(\rho) \sim \text{Normal}(0, \text{variance} = 1.5^2)$

where $Y_{j,t,\mathrm{a},r,e,m,w}$ is the estimated cases of the indicator in county j, year t, age group a, racial and ethnic population r, educational attainment e, marital status m, and state w, among a sample of n individuals with prevalence p. Model terms consist of:

- $\boldsymbol{\beta}_0$ is a global intercept;
- $\mathbf{X}_{1,j,t,r}$ is a vector of covariates for county j, year t, and racial and ethnic population r; $\boldsymbol{\beta}_1$ is the associated vector of fixed effect regression coefficients;

- $\gamma_{1,j,t',a',r}$ is a random intercept for county, racial and ethnic population, year spline basis, and age spline basis combinations;
- k_t is the number of time knots (three knots, evenly spaced from 2009 to 2019); t' is the corresponding index;
- k_a is the number of age knots, which varies by indicator due to age restrictions: three knots are placed at age groups 20–24, 50–54, and 85+, respectively, for indicators whose data coverage begins at age 18 (due to our grouping of age into 5-year bins, we drop data for ages 18–19 and begin the analysis at age 20 for these indicators); for disability indicators from ACS whose coverage begins at an earlier age, an additional fourth knot was placed at the youngest available age group (either age group 0–1 or 5–9); a' is the corresponding index;
- $S_{t'}(t)$ is the value of linear spline basis t' on year, evaluated at year t;
- $S_{a'}(a)$ is the value of linear spline basis a' on age, evaluated at age group a;
- $\gamma_{2,j}$ is a random intercept for county;
- $\gamma_{3,t,a,r,e,m|a\geq 20}$ is a random intercept for county, year, age group, racial and ethnic population, educational attainment, and marital status, evaluated only for ages 20+, because educational attainment and marital status were undefined for younger ages; this restriction only affected certain variables from ACS, for which predictions under age 20 were generated without inclusion of this model term;
- $\gamma_{4,t,a,r}$ is a random intercept for year, age group, and racial and ethnic population;
- and $\gamma_{5,r,e,m,w}$ is a random intercept for racial and ethnic population, educational attainment, marital status, and state.

For variables that were drawn from both BRFSS and Gallup, the preceding model form was adapted to include source-specific random and fixed effects and to remove age-time interactions except in the spatial effects (γ_1), due to incomplete overlap between the BRFSS and Gallup data in temporal coverage. We treat BRFSS as the gold standard source for which we generate model predictions, due to its improved temporal coverage relative to Gallup and its more stable racial and ethnic population definitions. These models used the following specification:

$$Y_{j,t,a,r,e,m,w,d} \sim \text{Binomial}\left(p_{j,t,a,r,e,m,w,d}, n_{j,t,a,r,e,m,w,d}\right) \tag{9}$$

$$\text{logit}\big(p_{j,t,a,r,e,m,w,d}\big) = \boldsymbol{\beta}_0 + \boldsymbol{\beta}_1 \cdot \boldsymbol{X}_{1,j,t,r} + \boldsymbol{\beta}_{2,d|d=\text{Gallup}} + \sum_{t'=1}^{k_t} \sum_{a'=1}^{k_a} \big(\gamma_{1,j,t',a',r} \cdot S_{t'}(t) \cdot S_{a'}(a)\big) \quad (10)$$

$$+ \gamma_{2,j} + \gamma_{5,r,e,m,w} + \gamma_{6,t,r,e,m} + \gamma_{7,a,r,e,m} + \gamma_{8,a,r,d} + \gamma_{9,t} + \gamma_{10,a} + \gamma_{11,r}$$

Priors:

$\gamma_6 \sim \text{LCAR: IID: LCAR: IID}(\rho_{6,t}, \rho_{6,e}, \sigma_6)$

$\gamma_7 \sim \text{LCAR: IID: LCAR: IID}(\rho_{7,a}, \rho_{7,e}, \sigma_7)$

$\gamma_8 \sim \text{LCAR: IID: IID}(\rho_{8,a}, \sigma_8)$

$\gamma_9 \sim \text{LCAR}(\rho_{9,t}, \sigma_9)$

$\gamma_{10} \sim \text{LCAR}(\rho_{10,a}, \sigma_{10})$

$\gamma_{11} \sim \text{IID}(\sigma_{11})$

Hyperpriors:

$\sigma^{-2} \sim \text{PC}(\sigma_0 = 5, \alpha = 0.05) \rightarrow \Pr(\sigma > 5) = 0.05$

$\text{logit}(\rho) \sim \text{Normal}(0, \text{variance} = 1.5^2)$

where $Y_{j,t,\text{a},r,e,m,w,d}$ is the estimated cases of the indicator in county j, year t, age group a, racial and ethnic population r, educational attainment e, marital status m, state w, and data source d. Model components are as described for equations 7 and 8, with the following additions (note the absence of γ_3 and γ_4 from equation 10; these random effects have been replaced here with γ_6 through γ_{11}):

- $\boldsymbol{\beta}_{2,d|d=\text{Gallup}}$ is a fixed effect for data from the Gallup survey;
- $\gamma_{6,t,r,e,m}$ is a random intercept for year, racial and ethnic population, educational attainment, and marital status (note that unlike γ_3 in equation 8, this term and $\gamma_{7,a,r,e,m}$ are not specified as conditional on age because these models all began at age 20+);
- $\gamma_{7,a,r,e,m}$ is a random intercept for age group, racial and ethnic population, educational attainment, and marital status;
- $\gamma_{8,a,r,d}$ is a random intercept for age group, racial and ethnic population, and data source;
- $\gamma_{9,t}$ is a random intercept for year;
- $\gamma_{10,a}$ is a random intercept for age group;
- and $\gamma_{11,r}$ is a random intercept for racial and ethnic population.

The following prior distributions were assigned for each random and fixed component in both of the above models (equations 7–8 and 9–10):

- Random effects on racial and ethnic population, marital status, state, or data source (ie, the relevant components of γ_1, γ_3, γ_4, γ_5, γ_6, γ_7, γ_8, and γ_{11}) were assumed to follow independent and identically distributed (IID) mean-0 Normal distributions, with variation specified by hyperparameters $\boldsymbol{\sigma}$.
- Effects on county, year, age group, or educational attainment (ie, the relevant components of γ_1, γ_2, γ_3, γ_4, γ_5, γ_6, γ_7, γ_8, γ_9, and γ_{10}) were assumed to follow conditional autoregressive distributions of the form described by Leroux, Lei, and Breslow (known as an LCAR prior).[19] LCAR priors correspond with the following full conditional distribution for each individual element, for example for effects by county j:

$$\gamma_j | \gamma_{k\sim j}, \sigma^2, \rho \sim \text{Normal}\left(\frac{\rho \cdot \sum_{k\sim j} \gamma_k}{n_j \cdot \rho + 1 - \rho}, \frac{\sigma^2}{n_j \cdot \rho + 1 - \rho}\right) \tag{11}$$

  where $k \sim j$ indicates the set of counties that are adjacent to county j and n_j is the number of counties in $k \sim j$. In this distribution, the σ^2 parameter controls the degree of spatial variation and the ρ parameter, which varies between 0 and 1, represents correlation between neighbors. Connections between counties were determined by geographic adjacency, with adjacency enforced manually for some geographically isolated locations to stabilise estimates: Nantucket Island (connected to Barnstable County, MA), Hawai'i (Honolulu County was connected to San Diego County, CA), and Alaska (Anchorage Municipality was joined to King County, WA). The analogous LCAR specification is used for effects on year, age, and education, with adjacency between consecutive years, age groups, and educational attainment categories (Less than High School, High School, Some College, BA or Higher). Unlike other model terms on age and year, γ_1 used conditional autoregressive distributions on age and year linear spline basis functions, rather than the full age and year indices.
- Random effects consisting of interactions among two or more dimensions (ie, γ_1, γ_3, γ_4, γ_5, γ_6, γ_7, and γ_8) were assumed to follow mean-0 multivariate Normal distributions with separable covariance structures defined via Kronecker products of the covariance matrices of a

combination of IID or LCAR priors. These random intercept terms were associated with hyperparameters for variation (σ).

Finally, hyperpriors were defined for the standard deviation (σ) and autocorrelation (ρ) hyperparameters:

- Penalised complexity (PC) priors were specified for the inverse variance ($1/\sigma^2$) of each random effect.[18] PC priors shrink toward a base model, which here is a marginal variance of 0. They are specified by setting the tail probability on each hyperparameter. We followed the recommendation by Fuglstad et al.,[18] selecting priors that satisfy $\Pr(\sigma > \sigma_0) = 0.05$ where σ_0 is between 2.5 and 40 times the expected true marginal standard deviation. Specifically, we set $\sigma_0 = 5; \Pr(\sigma > \sigma_0) = 0.05$.
- $\text{Normal}(0, \text{variance} = 1.5^2)$ priors were specified for the logit-transform of the correlation parameters (ρ).

These model specifications define the log-odds ratio of underlying prevalence (p) as a function of covariates and additional variation by county, year, age, racial and ethnic population, educational attainment, marital status, and data source. We selected the covariates included in this model—poverty rate, proportion born outside the US, income per capita, and population density—based on data availability and their previously observed associations with health. While county-level educational attainment may inform prevalence estimation, we did not use education covariates in these models because the models were already stratified by education. The fixed effects on covariates at the county level and covariates specific to county and racial and ethnic population capture relationships between each covariate and the prevalence of a given indicator.

Covariates do not explain all variation in prevalence across time, age, geography, racial and ethnic population, educational attainment, and marital status, or differences among data sources, so further random intercepts are included in the model to capture additional variation. For example, γ_1 allows for spatial (ie, between-county) variation in prevalence, shared across age, year, and racial and ethnic population. This particular random intercept incorporates a linear spline in the age and time dimensions to reduce computational complexity; the equivalent model for all age groups and years was found to be computationally infeasible. We chose the numbers of age and year knots to maximise flexibility while maintaining reasonable model runtimes. Although the splines in this random intercept are linear, we do

not assume that the time or age trends for $\text{logit}(p)$ are linear, as the contributions from the covariates and LCAR time effects in other model terms allow for non-linear variation.

We fit these models with the Template Model Builder (TMB) package[20] in R version 3.6.1[17], using an empirical Bayes approach. TMB calculates analytic approximations to the posterior distribution based on Laplace approximations. We used TMB rather than INLA—another alternative to Markov chain Monte Carlo (MCMC) methods and the tool that we used for the covariate models (section 3)—because TMB is substantially more flexible with respect to model specifications,[21] for example by enabling us to incorporate higher-order interaction terms and a bespoke process for disaggregation of input data (section 4.2).

4.2 Addressing data misalignment

Modeling variation in YLD rates at the detailed level of our study required predictors that were stratified to the same resolution as that desired for the YLD estimates. We therefore designed our indicator models to produce prevalence estimates for each indicator simultaneously for all combinations of 3 110 counties, 19 age groups (as applicable to a given covariate; eg, Gallup and BRFSS data were only available for age groups 20+), 11 years, five racial and ethnic populations, four educational attainment categories, three marital status categories, and two sexes. ACS, BRFSS, and Gallup release data with the demographic detail that we required for modelling YLD indicators in most dimensions, with three primary exceptions. Two of these exceptions were spatial: as previously described (section 1), ACS microdata are available at the level of PUMA rather than county, and the BRFSS data to which we had access were variously reported by county, CBSA, or state, necessitating the development of aggregate spatial units (PUMA-counties and CBSA-counties; section 2) whose boundaries corresponded with those of one or more combined counties. The third exception was related to age groups: starting in 2013, BRFSS top-coded age at 80, rather than 85 (the latter being the oldest age group in our analysis).

We applied a regression technique[22–24] that incorporates aggregated inputs during maximum likelihood optimization. In this approach, we calculate the linear predictor for each stratum based on the county-level covariates. We obtain the linear predictor for the aggregate unit by taking the population-weighted average of the linear predictors from each constituent stratum. The (negative log) likelihood is calculated

from the aggregated input data (cases), sample size, and linear predictor. This process is generalised as follows, for an aggregate stratum k consisting of g constituent strata:

$$Y_k \sim \text{Binomial}(p_k, n_k) \tag{12}$$

$$p_k = \frac{\sum_{i=1}^{g}(p_i \cdot w_i)}{\sum_{i=1}^{g} w_i} \tag{13}$$

$$n_k = \sum_{i=1}^{g} n_i \tag{14}$$

where w_i is the proportion of the population in k that is represented by stratum i. Population weights were derived from the post-stratification frame (section 4.3). Disaggregation regression is highly flexible in its application, being agnostic of the dimensionality of the aggregation within the input data. We used this approach for both spatially-aggregated and age-aggregated inputs in the YLD indicator models, as described above, but also employed it when fitting YLD models (section 5), for which the modelled outcome variable represents aggregated all-race and ethnicity estimates at the state level.

In our study, variation at the county level in models using BRFSS data was facilitated by county-level covariates, partial availability of county-level resolution in BRFSS in some states and years, and joint modelling with Gallup data (which uniformly had county identifiers) for some variables. For ACS models, county-level variation was provided by covariates. Simulation studies[25] have shown that the general approach to modelling aggregated input data described here performs better as sample size increases and as the number of child units per area decreases (ie, the degree of aggregation in the input data is reduced). The spatial units of our data inputs in the ACS and BRFSS models were often identical or similar in scope to counties, and the degree of bias and loss of precision stemming from the use of aggregate data was therefore likely to be relatively small in most locations. Similarly, the inclusion of aggregate age group 80+ data in the inputs to models for BRFSS variables likely had only minor impacts on accuracy and precision because BRFSS data for ages 80–84 and 85+ were available prior to 2013, and Gallup data for ages 85+ were available for indicators with data from both sources.

An important assumption underlying our approach to using aggregated survey data is that the combination of sampled individuals from constituent strata are population-representative of the larger aggregate stratum. This assumption requires that the distribution of survey respondents, appropriately

weighted to account for survey design and non-response, is proportional to the population sizes in the constituent strata. Such a scenario is unlikely to be realised precisely in most instances, with the consequent potential for bias to enter our model estimates. We acknowledge this as a limitation of our approach but argue that exclusion of the aggregate data is likely to introduce greater bias or loss of precision.

4.3 Post-stratification frame

We used multilevel regression with post-stratification (MrP)[26–28] to adjust for non-response bias in survey data. This procedure adjusts final model estimates by weighting modelled strata such that they have the same demographic distribution as the target population. While sample weights are commonly used to adjust for non-response bias in survey data, sample weights were calculated differently in our data sources (ACS, BRFSS, and Gallup) and even within the same data source over time. These inconsistencies in survey weights limit the comparability of temporal and combined trends by data source. Moreover, the survey weights were only adjusted to sub-state regions and states and were therefore not designed to adjust for sampling bias at the county level.

As previously described, we included educational attainment and marital status as dimensions in our indicator models. After model-fitting, we generated predictions at the full stratification present in the models, then collapsed predictions across educational attainment and marital status to derive final estimates by county, age, sex, year, and race and ethnicity. This aggregation was performed by taking the weighted average of the education and marital-specific estimates, using population estimates from the post-stratification frame as weights.

In the absence of county-level population data simultaneously stratified across all the dimensions of our final estimates, in addition to educational attainment and marital status, we needed to estimate the detailed population distribution (ie, the post-stratification frame) to be able to apply the MrP method. The post-stratification frame estimation method was adapted from Leeman et al.[29] This method, also referred to as MrP using synthetic poststratification (MrsP), was adopted with some technical alterations such as the use of raking to allow for the multi-dimensional split of population data.

The steps we used (iterative proportional fitting) to produce the post-stratification frame can be summarized as follows: (1) compiling and adjusting the population data to serve as raking margins; (2)

obtaining a raw initial population distribution; and (3) raking the initial distribution to the known population margins to obtain the post-stratification frame.

Compiling the raking margins: We obtained population estimates from ACS at the county level by age, sex, year, and each of the following variables separately: racial and ethnic population, marital status, and educational attainment. Additionally, we used the population distribution from IPUMS at the state and national levels stratified jointly by age, sex, racial and ethnic population, educational attainment, and marital status, by year. Of note, one condition for raking is that the marginal distributions should sum to the same total overall and across overlapping margins (eg age and sex). This was not the case for the marginal distributions we used, given differences in data sources, suppression rules at the county level, and data processing. Thus, we opted to scale the county level marginal distributions so that they sum to the state-level distribution across all overlapping variables (ie age, sex, racial and ethnic population, marital status, educational attainment, and year).

Obtaining an initial population distribution: We obtained an initial joint distribution frame by applying the same joint distribution observed at the state level to all counties in a state adjusted to the county population size by age, sex, and race.

Raking: We then raked the joint distribution iteratively by each of the county, state, and national population marginal distributions listed above, until convergence was achieved or after 40 iterations. Convergence was defined as having a maximum difference of 10^{-5} between the sum of the joint cells and each of the raking marginal distributions. The resulting frame had 5 224 800 strata per year and was vetted to ensure alignment with each of the marginal distributions in all years.

4.4 Hyperprior sensitivity analysis

We evaluated the impact of varying our prior assumptions using a range of alternative hyperparameter specifications for a selection of indicators (cognitive difficulty, asthma, and pain). These variables were selected because they provided a range of models with varying patterns of morbidity by age, sex, and geography, and represented differing combinations of data sources. These priors imply different degrees of variability in values for a given random effect. We tested the following penalised complexity (PC) specifications for variance parameters, for each of the selected indicators:

- $\sigma_0 = 1;\ \Pr(\sigma > \sigma_0) = 1.25 \times 10^{-3}, 0.05, 0.8$
- $\sigma_0 = 5;\ \Pr(\sigma > \sigma_0) = 1.25 \times 10^{-3}, 0.05, 0.8$

Separately, we also tested the influence of priors on the logit of the correlation parameters, ρ, adjusting both means and variances to evaluate different assumptions about the strength of the autocorrelation. We tested every combination of the following sets of priors on the mean and variance of ρ, applying them to all ρ parameters within a given model run:

- $\text{logit}(\rho) \sim \text{Normal}(\{0, 5\}, \text{variance} = \{0.1^2, 1.5^2\})$

Comparisons were made between point (mean) prevalence estimates for each county, year, age, sex, and race and ethnicity stratum. Model results were highly similar across parameterisations of the variance priors (Supplementary Figure 2), indicating a predominant influence of the input data on the model. Model predictions were also similar among the tested priors on ρ, although the model was more sensitive to strong priors on ρ (those with very low variance) than to the priors on σ_0, and showed greater variability in their differences from the base model. In the absence of *a priori* knowledge from other sources about appropriate degrees of autocorrelation by time or age, the weak priors that we use in the base model feel most appropriate, as they put greater emphasis on the information content of the model data.

5. YLD estimation

5.1 Cause hierarchy

We estimated YLD rates by county, year, age, sex, and racial and ethnic population for 16 groups of conditions that are collectively responsible for all causes of morbidity in GBD 2021 (Supplementary Table 7). These causes include a single umbrella cause for all communicable, maternal, neonatal, and nutritional disorders, which accounted for only 3.4% of YLDs in the USA in 2019; three categories of injuries; and 12 categories of non-communicable diseases such as cancers and musculoskeletal disorders.

5.2 Covariate selection

We used a wide range of covariates to model variation in YLD rates. As with the YLD indicator models, we included several sociodemographic covariates that we considered likely to be associated with morbidity and treated them as candidate predictors for every YLD cause. These covariates consisted of county, year, race and ethnicity-specific rates of educational attainment (separate covariates for High School graduate or higher, and for BA degree or higher), born outside the US, income per capita, and poverty, and county and year-specific population density (Supplementary Table 6). We also used our modelled YLD indicator

estimates as covariates in the YLD models, providing information at the level of stratification that we required for our final YLD estimates. We considered some indicators, such as pain and frequent activity limitation, to be general indicators of health and candidates for model inclusion for all YLD causes. The remaining indicators were only incorporated in models for causes that were closely related to the indicator; for example, asthma prevalence was used as a predictor only in the model for respiratory diseases. Finally, we included estimates of YLLs specific to cause, county, year, race and ethnicity, sex, and age combinations[2] to leverage relationships between morbidity and mortality for a given cause.

Given the large number of potential covariates in these models, we tested for multicollinearity by calculating Variance Inflation Factors (VIF)[30] for each covariate in each modelled cause. As our goal in this study was to predict YLD rates and ultimately HALE, rather than making inferences about the direction or scale of associations between outcomes and covariates, our primary concern was in producing stable model estimates. In this context, multicollinearity is less concerning than had we been attempting inference, but strong multicollinearity could still lead to unreasonably extreme estimates. We therefore set a high threshold for VIF, using a value of 10. No covariates yielded a VIF value above 10 in any YLD model, so all candidate covariates were retained.

5.3 Model specification

YLD rates were estimated using a modelling approach similar to those used for YLD indicators, but with some key differences. First, the outcome measure represented state-level YLD rate estimates from GBD 2021 for all racial and ethnic populations combined, by age group, rather than race and ethnicity-specific county-level (or close to county-level) survey observations. Since this outcome variable was a modelled quantity rather than a sample from a survey (and hence lacked associated sample sizes), but was a proportion bounded by 0 and 1, we assumed a Normal distribution on the logit-transformed YLD rates. We modelled county-level and racial and ethnic population-specific YLD rates using the disaggregation approach previously described (section 4.2). The specificity of the life tables, YLL estimates and other covariates (section 3) provided the variability with which to estimate higher-resolution YLD rates than are available from the GBD 2021 results. Second, to better capture the association between covariates and geographic variation in YLD rates, we did not include state-level random effects, as these would have absorbed much of the geographic variability and reduced the explanatory power of the covariates that capture county-level variation. Finally, the model lacks terms for educational attainment (except as

covariates) and marital status as these dimensions are neither present in the outcome variable nor required for our final estimates.

Models were fit separately by sex for each of the 16 modelled causes, using the following specification:

$$\text{logit}(R_{k,t,a}) \sim \text{Normal}(\text{logit}(\mu_{k,t,a}), \sigma^2) \quad (15)$$

$$\mu_{k,t,a} = \frac{\sum_{j\in k,r}(\mu_{j,t,a,r} \cdot w_{j,t,a,r})}{\sum_{j\in k,r} w_{j,t,a,r}} \quad (16)$$

$$\text{logit}(\mu_{j,t,a,r}) = \eta_{j,t,a,r} = \boldsymbol{\beta}_0 + (\boldsymbol{\beta}_1 + \gamma_{1,a}) \cdot \boldsymbol{X}_{j,t,a,r} \cdot \boldsymbol{U}_a + \gamma_{2,t,a} \quad (17)$$

Priors:

$\gamma_1 \sim \text{IID}(\sigma_1)$

$\gamma_2 \sim \text{LCAR: LCAR}(\rho_{2,t}, \rho_{2,a}, \sigma_2)$

Hyperpriors:

$\sigma^{-2} \sim \text{PC}(\sigma_0 = 5, \alpha = 0.05) \rightarrow \Pr(\sigma > 5) = 0.05$

$\text{logit}(\rho) \sim \text{Normal}(0, \text{variance} = 1.5^2)$

where $R_{k,t,a}$ is the YLD rate for state *k*, year *t*, and age group *a* from GBD 2021. We assumed a Gaussian distribution for logit-transformed YLD rates, with variance σ^2. The YLD rate for each state *k*, year *t*, and age group *a* was modelled as the population-weighted average of YLD rate estimates for the counties *j* and racial and ethnic populations *r* within that state-year-age stratum, with population weights w. Model terms consist of:

- $\boldsymbol{\beta}_0$ is a global intercept;
- $\boldsymbol{\beta}_1$ is a fixed covariate effect;
- $\mathbf{X}_{1,j,t,a,r}$ is a vector of covariates for county j, year t, age group *a*, and racial and ethnic population r; covariate effects are modelled via a combination of two terms:
    - $\boldsymbol{\beta}_1$ is the associated vector of fixed effect regression coefficients;
    - $\gamma_{1,a}$ is an IID random intercept for age, allowing covariate effects to vary by age;
- $\boldsymbol{U}_a$ is an indicator specifying whether a covariate is relevant for a given age group, as some covariates were only available for a subset of modelled ages; covariates are excluded from the linear predictor and predictions for ages that they do not cover;
- and $\gamma_{2,t,a}$ is a random intercept for year and age with LCAR priors.

5.4 Uncertainty propagation

Nearly all covariates and outcome variables in the YLD models are derived from modelled estimates; the exception is population density, which is based on point estimates of modelled population sizes from the Census Bureau. To propagate uncertainty from the modelled inputs throughout the YLD estimation pipeline, we ran 50 models for each YLD cause, each time using separate draws from the posterior distributions of the covariates and outcomes, with independence of draws assumed among outcomes and covariates. We chose to use 50 draws as a compromise between computation time and the accuracy of the variance approximation. To aid model convergence, an initial model was run for each cause using the point estimates of each covariate and outcome to provide starting values for parameters. Twenty draws were taken from every fitted model to obtain 1000 total draws for each YLD cause.

5.5 Hyperprior sensitivity analysis

We evaluated the impact of alternative hyperparameter specifications for a selection of YLD causes: musculoskeletal disorders; transportation injuries; and neonatal, maternal, nutritional, and communicable diseases. These causes were selected because they provided a range of models with varying patterns of morbidity by age, sex, and geography, and represented each of the three major subdivisions of non-fatal causes in the GBD 2021 cause hierarchy. We tested the following penalised complexity (PC) specifications for variance parameters in the YLD models, for each of the selected causes:

- $\sigma_0 = 1;\ \Pr(\sigma > \sigma_0) = 1.25 \times 10^{-3}, 0.05, 0.8$
- $\sigma_0 = 5;\ \Pr(\sigma > \sigma_0) = 1.25 \times 10^{-3}, 0.05, 0.8$

Separately, we also tested the influence of priors on the logit of the correlation parameters, ρ, adjusting both means and variances to evaluate different assumptions about the strength of the autocorrelation. We tested every combination of the following sets of mean and variance priors, applying them to all ρ parameters within a given model run:

- $\text{logit}(\rho) \sim \text{Normal}(\{0, 5\}, \text{variance} = \{0.1^2, 1.5^2\})$

Supplementary Figure 3 shows the absolute and relative differences in county-level estimates derived from models using these alternate prior specifications, compared with the main model. Comparisons were made between point estimates of prevalence returned from TMB. Model results were highly similar across parameterisations of the variance priors, indicating low sensitivity to these priors and a

predominant influence of the input data. Model estimates were sensitive to strict priors on the autocorrelation parameters, in particular with the $\mathrm{Normal}(0, \mathrm{variance} = 0.1^2)$ prior, which represents a strong prior that ρ is close to 0.5—ie, only moderate correlation. However, such a strong prior is not suggested by any current *a priori* knowledge of the degree of correlation among YLD rates by age or time, and we feel confident that vague priors on ρ are appropriate for our models; estimates were similar between the two model runs that used autocorrelation priors with high variance.

5.6 Calibration

As the final steps in our estimation of YLD rates, we aggregated cause-specific YLD rates to produce all-cause (total) YLD rate estimates by county, year, age, sex, and racial and ethnic population. We then calibrated these estimates to match the state-specific all-cause YLD rate estimates from GBD 2021 via one-dimensional raking.[31] Although we had used YLD estimates from GBD as outcomes in our models, our estimates did not exactly match those from GBD due to the absence of state-level random effects in the models, and other factors. We therefore calibrated our estimates to conform to the state-level variation present in GBD to maintain consistency with that project and to leverage information provided by the additional data sources used by GBD, such as claims data and major survey series that we did not directly include in our models.

6. HALE estimation

We calculated healthy life expectancy (HALE) using the methods employed by GBD 2021,[32] using our all-cause YLD estimates to adjust life tables estimated by a study of county, race, and ethnicity-specific mortality.[2] This approach uses Sullivan's method, which is detailed in the GBD capstone on HALE[32] but reproduced here in brief. HALE was calculated using the following equations:

$$nLx_{adjusted} = nLx \cdot (1 - YLDs) \quad (18)$$

$$Tx_{adjusted,i} = \sum_{i}^{t} nLx_{adjusted} \quad (19)$$

$$HALE = \frac{Tx_{adjusted}}{lx} \quad (20)$$

where *nLx* is total person-years lived between age *x* and age *x* + *n*; *YLDs* are YLD rates (YLDs per capita); *Tx* is person-years lived above age *x*; and *i* is the age group currently being calculated, *t* being the terminal

age group. Although we estimated HALE for each age group that we modelled, we report results only for HALE at birth (age 0).

References

1 Dwyer-Lindgren L, Kendrick P, Kelly YO, *et al.* Life expectancy by county, race, and ethnicity in the USA, 2000–19: a systematic analysis of health disparities. *Lancet* 2022; **400**: 25–38.

2 Dwyer-Lindgren L, Kendrick P, Kelly YO, *et al.* Cause-specific mortality by county, race, and ethnicity in the USA, 2000–19: a systematic analysis of health disparities. *Lancet* 2023; **402**: 1065–82.

3 National Center for Health Statistics, Centers for Disease Control and Prevention, US Census Bureau. United States Bridged-Race Intercensal Population Estimates 2000-2009. 2012; published online Oct 30. https://www.cdc.gov/nchs/nvss/bridged_race.htm (accessed Dec 12, 2022).

4 National Center for Health Statistics, Centers for Disease Control and Prevention, US Census Bureau. United States Vintage 2019 Bridged-Race Postcensal Population Estimates 2010-2019. 2020. https://www.cdc.gov/nchs/nvss/bridged_race.htm (accessed July 20, 2020).

5 Office of Management and Budget. OMB Directive 15: Race and Ethnic Standards for Federal Statistics and Administrative Reporting. 1977. https://wonder.cdc.gov/wonder/help/populations/bridged-race/directive15.html (accessed Sept 17, 2020).

6 National Center for Health Statistics (U.S.), editor. U.S. census 2000 population with bridged categories. Hyattsville, Md: National Center for Health Statistics, 2003.

7 Centers for Disease Control and Prevention (CDC). Centers for Disease Control and Prevention (CDC). United States Behavioral Risk Factor Surveillance System, 2009–2019. 2021.

8 Gallup, Sharecare. United States - Gallup Daily Sharecare Well-being Index Track 2009–2016. 2017. https://news.gallup.com/poll/106756/galluphealthways-wellbeing-index.aspx (accessed Jan 24, 2024).

9 United States Census Bureau (USCB). United States American Community Survey 2009–2019. 2021.

10 Liebler, Carolyn A. Building New Bridges: Developing and Disseminating a Simplified Race/Ethnicity Measure for Working with Complex or Contradictory Race Data. *Minn Popul Cent Work Pap Ser* 2022. DOI:10.18128/MPC2022-02.

11 Ferrari AJ, Santomauro DF, Aali A, *et al.* Global incidence, prevalence, years lived with disability (YLDs), disability-adjusted life-years (DALYs), and healthy life expectancy (HALE) for 371 diseases and injuries in 204 countries and territories and 811 subnational locations, 1990–2021: a systematic analysis for the Global Burden of Disease Study 2021. *The Lancet* 2024; **403**: 2133–61.

12 US Census Bureau. Instructions for Applying Statistical Testing to American Community Survey Data. 2019. https://www2.census.gov/programs-surveys/acs/tech_docs/statistical_testing/2019_Instructions_for_Stat_Testing_ACS.pdf (accessed Dec 17, 2020).

13 US Census Bureau CHS. Overview - History - U.S. Census Bureau. https://www.census.gov/history/www/through_the_decades/overview/ (accessed Feb 27, 2023).

14 Franco C, Little RJA, Louis TA, Slud EV. Comparative Study of Confidence Intervals for Proportions in Complex Sample Surveys. *J Surv Stat Methodol* 2019; **7**: 334–64.

15 Warton DI, Hui FKC. The arcsine is asinine: the analysis of proportions in ecology. *Ecology* 2011; **92**: 3–10.

16 Rue H, Martino S, Chopin N. Approximate Bayesian inference for latent Gaussian models by using integrated nested Laplace approximations. *J R Stat Soc Ser B Stat Methodol* 2009; **71**: 319–92.

17 R: A language and environment for statistical computing. R Foundation for Statistical Computing, Vienna, Austria. URL https://www.R-project.org/. 2022.

18 Fuglstad G-A, Simpson D, Lindgren F, Rue H. Constructing priors that penalize the complexity of Gaussian random fields. *J Am Stat Assoc* 2019; **114**: 445–52.

19 Leroux BG, Lei X, Breslow N. Estimation of Disease Rates in Small Areas: A new Mixed Model for Spatial Dependence. In: Halloran ME, Berry D, eds. Statistical Models in Epidemiology, the Environment, and Clinical Trials. New York, NY: Springer New York, 2000: 179–91.

20 Kristensen K, Nielsen A, Berg CW, Skaug H, Bell BM. TMB: Automatic Differentiation and Laplace Approximation. *J Stat Softw* 2016; **70**: 1–21.

21 Osgood-Zimmerman A, Wakefield J. A Statistical Introduction to Template Model Builder: A Flexible Tool for Spatial Modeling. 2021; published online March 17. DOI:10.48550/arXiv.2103.09929.

22 Lucas TCD, Nandi AK, Chestnutt EG, *et al.* Mapping malaria by sharing spatial information between incidence and prevalence data sets. *J R Stat Soc Ser C Appl Stat* 2021; **70**: 733–49.

23 Wilson K, Wakefield J. Pointless spatial modeling. *Biostatistics* 2020; **21**: e17–32.

24 Dwyer-Lindgren L, Squires ER, Teeple S, *et al.* Small area estimation of under-5 mortality in Bangladesh, Cameroon, Chad, Mozambique, Uganda, and Zambia using spatially misaligned data. *Popul Health Metr* 2018; **16**: 13.

25 Utazi CE, Thorley J, Alegana VA, *et al.* A spatial regression model for the disaggregation of areal unit based data to high-resolution grids with application to vaccination coverage mapping. *Stat Methods Med Res* 2019; **28**: 3226–41.

26 Hanretty C. An Introduction to Multilevel Regression and Post-Stratification for Estimating Constituency Opinion. *Polit Stud Rev* 2020; **18**: 630–45.

27 Ghitza Y, Gelman A. Deep Interactions with MRP: Election Turnout and Voting Patterns Among Small Electoral Subgroups: DEEP INTERACTIONS WITH MRP. *Am J Polit Sci* 2013; **57**: 762–76.

28 Gelman A, Lax J, Phillips J, Gabry J, Trangucci R. Using Multilevel Regression and Poststratification to Estimate Dynamic Public Opinion. *Unpubl Manuscr Columbia Univ* 2018; : 48.

29 Leemann L, Wasserfallen F. Extending the Use and Prediction Precision of Subnational Public Opinion Estimation. *Am J Polit Sci* 2017; **61**: 1003–22.

30 Thompson CG, Kim RS, Aloe AM, Becker BJ. Extracting the Variance Inflation Factor and Other Multicollinearity Diagnostics from Typical Regression Results. *Basic Appl Soc Psychol* 2017; **39**: 81–90.

31 Dwyer-Lindgren L, Bertozzi-Villa A, Stubbs RW, *et al.* US county-level trends in mortality rates for major causes of death, 1980-2014. *JAMA* 2016; **316**: 2385–401.

32 Wang H, Abbas KM, Abbasifard M, *et al.* Global age-sex-specific fertility, mortality, healthy life expectancy (HALE), and population estimates in 204 countries and territories, 1950–2019: a comprehensive demographic analysis for the Global Burden of Disease Study 2019. *Lancet* 2020; **396**: 1160–203.

Supplementary Tables

Supplementary Table 1. GATHER Checklist

Item #	Checklist item	Description of Compliance
Objectives and funding		
1	Define the indicator(s), populations (including age, sex, and geographic entities), and time period(s) for which estimates were made.	Abstract, Introduction, Methods
2	List the funding sources for the work.	Article Information
Data Inputs		
For all data inputs from multiple sources that are synthesized as part of the study:		
3	Describe how the data were identified and how the data were accessed.	Methods
4	Specify the inclusion and exclusion criteria. Identify all ad-hoc exclusions.	Methods
5	Provide information on all included data sources and their main characteristics. For each data source used, report reference information or contact name/institution, population represented, data collection method, year(s) of data collection, sex and age range, diagnostic criteria or measurement method, and sample size, as relevant.	Methods, Supplementary Methods, Supplementary Tables 4–6
6	Identify and describe any categories of input data that have potentially important biases (eg based on characteristics listed in item 5).	Methods
For data inputs that contribute to the analysis but were not synthesized as part of the study:		
7	Describe and give sources for any other data inputs.	N/A
For all data inputs:		
8	Provide all data inputs in a file format from which data can be efficiently extracted (eg a spreadsheet rather than a PDF), including all relevant meta-data listed in item 5. For any data inputs that cannot be shared because of ethical or legal reasons, such as third-party ownership, provide a contact name or the name of the institution that retains the right to the data.	GHDx link
Data analysis		
9	Provide a conceptual overview of the data analysis method. A diagram may be helpful.	Methods, Supplementary Figure 1
10	Provide a detailed description of all steps of the analysis, including mathematical formulae. This description should cover, as relevant, data cleaning, data pre-processing, data adjustments and weighting of data sources, and mathematical or statistical model(s).	Methods, Supplementary Methods
11	Describe how candidate models were evaluated and how the final model(s) were selected.	Supplementary Methods
12	Provide the results of an evaluation of model performance, if done, as well as the results of any relevant sensitivity analysis.	N/A
13	Describe methods for calculating uncertainty of the estimates. State which sources of uncertainty were, and were not, accounted for in the uncertainty analysis.	Methods, Supplementary Methods

Item #	Checklist item	Description of Compliance
14	State how analytic or statistical source code used to generate estimates can be accessed.	GHDx link
Results and Discussion		
15	Provide published estimates in a file format from which data can be efficiently extracted.	GHDx link
16	Report a quantitative measure of the uncertainty of the estimates (e.g. uncertainty intervals).	Results, GHDx link
17	Interpret results in light of existing evidence. If updating a previous set of estimates, describe the reasons for changes in estimates.	Introduction, Discussion
18	Discuss limitations of the estimates. Include a discussion of any modelling assumptions or data limitations that affect interpretation of the estimates.	Discussion

Supplementary Table 2. Counties combined to create historically stable units of analysis

State	Group	Counties (FIPS code)
Alaska	1	Chugach Census Area (2063), Copper River Census Area (2066), Valdez-Cordova Census Area (2261)*
Alaska	2	Kusilvak Census Area (2158), Wade Hampton Census Area (2270)*
	3	Kobuk Census Area (2140)*, Northwest Arctic Borough (2188)
	4	Aleutian Islands Census Area (2010)*, Aleutians East Borough (2013), Aleutians West Census Area (2016)
	5	Dillingham Census Area (2070), Lake and Peninsula Borough (2164)
	6	Denali Borough (2068), Yukon-Koyukuk Census Area (2290)
	7	Hoonah-Angoon Census Area (2105), Skagway Municipality (2230), Skagway-Yakutat-Angoon Census Area (2231)*, Skagway-Hoonah-Angoon Census Area (2232)*, Yakutat City and Borough (2282)
	8	Ketchikan Gateway Borough (2130), Petersburg Borough (2195), Prince of Wales-Hyder Census Area (2198), Prince of Wales-Outer Ketchikan Census Area (2201)*, Wrangell City and Borough (2275), Wrangell-Petersburg Census Area (2280)*
Arizona	1	La Paz County (4012), Yuma County (4027)
Colorado	1	Adams County (8001), Arapahoe County (8005), Boulder County (8013), Broomfield County (8014), Denver County (8031), Jefferson County (8059), Weld County (8123)
Florida	1	Dade County (12025)*, Miami-Dade County (12086)
Hawaii	1	Kalawao County (15005), Maui County (15009)
Maryland	1	Montgomery County (24031), Prince George's County (24033)
Montana	1	Park County (30067), Yellowstone National Park (30113)*
New Mexico	1	Cibola County (35006), Valencia County (35061)
South Dakota	1	Oglala Lakota County (46102), Shannon County (46113)*
	2	Jackson County (46071), Washabaugh County (46131)*
Virginia	1	Fairfax County (51059), Fairfax City (51600)
	2	Rockingham County (51165), Harrisonburg City (51660)
	3	James City County (51095), Williamsburg City (51830)
	4	Prince William County (51153), Manassas City (51683), Manassas Park City (51685)
	5	Rockbridge County (51163), Buena Vista City (51530)
	6	Spotsylvania County (51177), Fredericksburg City (51630)
	7	Augusta County (51015), Staunton City (51790), Waynesboro City (51820)
	8	Pittsylvania County (51143), Danville City (51590)
	9	Greensville County (51081), Emporia City (51595)
	10	Albemarle County (51003), Charlottesville City (51540)
	11	Bedford County (51019), Bedford City (51515)*
	12	Halifax County (51083), South Boston City (51780)*
	13	Southampton County (51175), Franklin City (51620)
	14	Alleghany County (51005), Clifton Forge City (51560)*
	15	York County (51199), Newport News City (51700)

*County no longer exists due to boundary or name change. FIPS = Federal Information Processing Standards.

Supplementary Table 3. Indicators of non-fatal burden

Outcome	Definition	Data sources	Spatial resolution	Data years used	Age range	Sample Size[a]
Cognitive Difficulty	Responded “yes” to: “Because of a physical, mental, or emotional condition, does this person have serious difficulty concentrating, remembering, or making decisions?”	ACS	PUMA-county	2009, 2011–2019	5+	29805968
Personal Care Difficulty	Responded “yes” to: “Does this person have difficulty dressing or bathing?”	ACS	PUMA-county	2009, 2011–2019	5+	29805968
Physical Difficulty	Responded “yes” to: “Does this person have serious difficulty walking or climbing stairs?”	ACS	PUMA-county	2009, 2011–2019	5+	29805968
Mobility Limitation	Responded “yes” to: “Because of a physical, mental, or emotional condition, does this person have difficulty doing errands alone such as visiting a doctor's office or shopping?”	ACS	PUMA-county	2009, 2011–2019	16+	23936306
Vision or Hearing Disability	Responded “yes” to at least one of the following questions: “Is this person deaf or does he/she have serious difficulty hearing? [yes/no]”, “Is this person blind or does he/she have serious difficulty seeing even when wearing glasses?”	ACS	PUMA-county	2009, 2011–2019	0+	30496313
Frequent Activity Limitation	Reported ≥14 days for the following question: “During the past 30 days, for about how many days did poor physical or mental health keep you from doing your usual activities, such as self-care, work, or recreation?”	BRFSS	CBSA-county	2011–2016, 2018-2019	18+	3452089
	Reported ≥14 days for the following question: “During the past 30 days, for about how many days did poor health keep you from doing your usual activities?”	Gallup	County	2009–2016	18+	1924555
Asthma	Responded “yes” to: “Has a doctor, nurse, or other health professional EVER told you that you had any of the following? (Ever told) you had asthma?”	BRFSS	CBSA-county	2011–2019	18+	3482885
	Responded “yes” to: “Have you ever been told by a physician or nurse that you have any of the following, or not? How about *asthma*?”	Gallup	County	2009–2016	18+	1776422

Depression	Responded "yes" to: "Has a doctor, nurse, or other health professional EVER told you that you had any of the following? (Ever told) you have a depressive disorder (including depression, major depression, dysthymia, or minor depression)?"	BRFSS	CBSA-county	2011–2019	18+	3477309
	Responded "yes" to: "Have you ever been told by a physician or nurse that you have any of the following, or not? How about *depression*?"	Gallup	County	2009–2016	18+	1940591
Diabetes	Responded "yes" to: "Has a doctor, nurse, or other health professional EVER told you that you had any of the following? (Ever told) you have diabetes?"	BRFSS	CBSA-county	2011–2019	18+	3486886
	Responded "yes" to: "Have you ever been told by a physician or nurse that you have any of the following, or not? How about *diabetes*?"	Gallup	County	2009–2016	18+	1940787
Arthritis	Responded "yes" to: "Has a doctor, nurse, or other health professional EVER told you that you had any of the following? (Ever told) you have some form of arthritis, rheumatoid arthritis, gout, lupus, or fibromyalgia?"	BRFSS	CBSA-county	2009–2019	18+	3894252
COPD	Responded "yes" to: "Has a doctor, nurse, or other health professional EVER told you that you had any of the following? (Ever told) you have (COPD) chronic obstructive pulmonary disease, emphysema or chronic bronchitis?"	BRFSS	CBSA-county	2009–2019	18+	3896482
Severe Tooth Loss	Reported 6 or more teeth removed (including responses of "all" teeth removed) in response to: "How many of your permanent teeth have been removed because of tooth decay or gum disease? Include teeth lost to infection, but do not include teeth lost for other reasons, such as injury or orthodontics."	BRFSS	CBSA-county	2010–2018 (even years only)	18+	1707720
Pain	Responded "yes" to: "Did you experience the following feelings during A LOT OF THE DAY yesterday? How about *physical pain*?"	Gallup	County	2009–2016	18+	1941395
Stress	Responded "yes" to: "Did you experience the following feelings during A LOT OF THE DAY yesterday? How about *stress*?"	Gallup	County	2009–2016	18+	1941619

Worry	Responded “yes” to: “Did you experience the following feelings during A LOT OF THE DAY yesterday? How about *worry*?”	Gallup	County	2009–2016	18+	1941786

[a]Across all years of data used.

Supplementary Table 4. Health indicator survey data sources

Title	Years covered	Citation
American Community Survey	2009–2014	Minnesota Population Center, US Census Bureau. United States American Community Survey 2009–2014 from the Integrated Public Use Microdata Series, International: [Machine-readable database]. Minneapolis: University of Minnesota, 2013
American Community Survey	2015–2017	United States Census Bureau, Minnesota Population Center. United States American Community Survey 2015–2017 from the Integrated Public Use Microdata Series, International. Version 7.1 [dataset]. Minneapolis, MN: IPUMS, 2018. https://doi.org/10.18128/D020.V7.1
American Community Survey	2018	Steven Ruggles, Sarah Flood, Ronald Goeken, Josiah Grover, Erin Meyer, Jose Pacas and Matthew Sobek. IPUMS USA: Version 10.0 [dataset]. Minneapolis, MN: IPUMS, 2020. https://doi.org/10.18128/D010.V10.0.
American Community Survey	2019	Steven Ruggles, Sarah Flood, Ronald Goeken, Megan Schouweiler and Matthew Sobek. IPUMS USA: Version 12.0 [dataset]. Minneapolis, MN: IPUMS, 2022. https://doi.org/10.18128/D010.V12.0
Alaska Behavioral Risk Factor Surveillance System	2014–2016, 2018–2019	Alaska Department of Health, Division of Public Health, Section of Chronic Disease Prevention and Health Promotion, Centers for Disease Control and Prevention (CDC). United States - Alaska Behavioral Risk Factor Surveillance System 2014–2016, 2018–2019. Anchorage, AK, United States of America: Alaska Department of Health and Social Services, 2015–2017, 2019–2020.
Arizona Behavioral Risk Factor Surveillance System	2011–2016, 2018–2019	Arizona Department of Health Services (ADHS), Centers for Disease Control and Prevention (CDC). United States - Arizona Behavioral Risk Factor Surveillance System 2011–2016, 2018–2019. Phoenix, AZ, United States of America: Arizona Department of Health Services (ADHS).
Arkansas Behavioral Risk Factor Surveillance System	2011–2016, 2018–2019	Arkansas Department of Health, Centers for Disease Control and Prevention (CDC). United States - Arkansas Behavioral Risk Factor Surveillance System 2011–2016, 2018–2019. Little Rock, United States of America: Arkansas Department of Health.
California Behavioral Risk Factor Surveillance System	2013–2016, 2018–2019	California Behavioral Risk Factor Surveillance System Program, California Department of Public Health, Centers for Disease Control and Prevention (CDC), Public Health Survey Research Program

Title	Years covered	Citation
		(California State University, Sacramento). United States - California Behavioral Risk Factor Surveillance System 2013–2016, 2018–2019.
Connecticut Behavioral Risk Factor Surveillance System	2011–2016, 2018–2019	Centers for Disease Control and Prevention (CDC), Connecticut Department of Public Health. United States - Connecticut Behavioral Risk Factor Surveillance System 2011–2016, 2018–2019.
Delaware Behavioral Risk Factor Surveillance System	2011–2016, 2018–2019	Delaware Division of Public Health (United States), Delaware Health and Social Services. United States - Delaware Behavioral Risk Factor Surveillance System 2011–2016, 2018–2019.
Florida Behavioral Risk Factor Surveillance System	2013, 2016	Centers for Disease Control and Prevention (CDC), Florida Department of Health. United States - Florida Behavioral Risk Factor Surveillance System 2013, 2016. Tallahassee, FL, United States of America: Florida Department of Health.
Hawaii Behavioral Risk Factor Surveillance System	2011–2016, 2018–2019	Centers for Disease Control and Prevention (CDC), Hawaii State Department of Health. United States - Hawaii Behavioral Risk Factor Surveillance System 2011–2016, 2018–2019.
Illinois Behavioral Risk Factor Surveillance System	2011–2016, 2018–2019	Centers for Disease Control and Prevention (CDC), Illinois Department of Public Health. United States - Illinois Behavioral Risk Factor Surveillance System 2011–2016, 2018–2019.
Kansas Behavioral Risk Factor Surveillance System	2011–2016, 2018–2019	Centers for Disease Control and Prevention (CDC), Kansas Department of Health and Environment. United States - Kansas Behavioral Risk Factor Surveillance System 2011–2016, 2018–2019.
Massachusetts Behavioral Risk Factor Surveillance System	2011–2016, 2018–2019	Centers for Disease Control and Prevention (CDC), Massachusetts Executive Office of Health and Human Services (EOHHS). United States - Massachusetts Behavioral Risk Factor Surveillance System 2011–2016, 2018–2019.
Mississippi Behavioral Risk Factor Surveillance System	2011–2012	Centers for Disease Control and Prevention (CDC), Mississippi State Department of Health. United States - Mississippi Behavioral Risk Factor Surveillance System 2011–2012. Jackson, MS, United States of America: Mississippi State Department of Health.
New Mexico Behavioral Risk Factor Surveillance System	2011–2016, 2018–2019	Centers for Disease Control and Prevention (CDC), New Mexico Department of Health. United States - New Mexico Behavioral Risk Factor Surveillance System 2011–2016, 2018–2019.
New York Behavioral Risk Factor Surveillance System	2011–2016, 2018–2019	Centers for Disease Control and Prevention (CDC), New York State Department of Health. United

Title	Years covered	Citation
		States - New York Behavioral Risk Factor Surveillance System 2011–2016, 2018–2019.
Ohio Behavioral Risk Factor Surveillance System	2013–2016, 2018–2019	Centers for Disease Control and Prevention (CDC), Ohio Department of Health. United States - Ohio Behavioral Risk Factor Surveillance System 2013–2016, 2018–2019.
Pennsylvania Behavioral Risk Factor Surveillance System	2011–2016, 2018–2019	Centers for Disease Control and Prevention (CDC), Pennsylvania Department of Health. United States - Pennsylvania Behavioral Risk Factor Surveillance System 2011–2016, 2018–2019.
South Dakota Behavioral Risk Factor Surveillance System	2011–2015, 2018–2019	Centers for Disease Control and Prevention (CDC), South Dakota Department of Health. United States - South Dakota Behavioral Risk Factor Surveillance System 2011–2015, 2018–2019.
Texas Behavioral Risk Factor Surveillance System	2011–2016, 2018–2019	Centers for Disease Control and Prevention (CDC), Texas Department of State Health Services. United States - Texas Behavioral Risk Factor Surveillance System 2011–2016, 2018–2019.
Vermont Behavioral Risk Factor Surveillance System	2011–2016, 2018–2019	Centers for Disease Control and Prevention (CDC), Vermont Department of Health. United States - Vermont Behavioral Risk Factor Surveillance System 2011–2016, 2018–2019.
Virginia Behavioral Risk Factor Surveillance System	2011–2016, 2018–2019	Centers for Disease Control and Prevention (CDC), Virginia Department of Health (United States). United States - Virginia Behavioral Risk Factor Surveillance System 2011–2016, 2018–2019.
Washington Behavioral Risk Factor Surveillance System	2011	Washington State Department of Health, Center for Health Statistics, Behavioral Risk Factor Surveillance System, supported in part by the Centers for Disease Control and Prevention, Cooperative Agreement U58/SO000047-1 (2011).
Washington Behavioral Risk Factor Surveillance System	2012	Washington State Department of Health, Center for Health Statistics, Behavioral Risk Factor Surveillance System, supported in part by the Centers for Disease Control and Prevention, Cooperative Agreement U58/SO000047-2 (2012).
Washington Behavioral Risk Factor Surveillance System	2013	Washington State Department of Health, Center for Health Statistics, Behavioral Risk Factor Surveillance System, supported in part by the Centers for Disease Control and Prevention, Cooperative Agreement U58/SO000047-3 and 3U58SO000047-02W1 (2013).
Washington Behavioral Risk Factor Surveillance System	2014	Washington State Department of Health, Center for Health Statistics, Behavioral Risk Factor Surveillance System, supported in part by the Centers for

Title	Years covered	Citation
		Disease Control and Prevention, Cooperative Agreement U58/SO000047-4 and 3U58SO000047-03W1 (2014).
Washington Behavioral Risk Factor Surveillance System	2015	Washington State Department of Health, Center for Health Statistics, Behavioral Risk Factor Surveillance System, supported in part by the Centers for Disease Control and Prevention, Cooperative Agreement NU58/DP006066-01 (2015).
Washington Behavioral Risk Factor Surveillance System	2016	Washington State Department of Health, Center for Health Statistics, Behavioral Risk Factor Surveillance System, supported in part by the Centers for Disease Control and Prevention, Cooperative Agreement NU58/DP006066-02 (2016).
Washington Behavioral Risk Factor Surveillance System	2018	Washington State Department of Health, Center for Health Statistics, Behavioral Risk Factor Surveillance System, supported in part by the Centers for Disease Control and Prevention, Cooperative Agreement NU58/DP006066-04 (2018).
Washington Behavioral Risk Factor Surveillance System	2019	Washington State Department of Health, Center for Health Statistics, Behavioral Risk Factor Surveillance System, supported in part by the Centers for Disease Control and Prevention, Cooperative Agreement U58/DP006066-05 (2019).
Wisconsin Behavioral Risk Factor Surveillance System	2011–2016, 2018–2019	Centers for Disease Control and Prevention (CDC), Wisconsin Department of Health Services. United States - Wisconsin Behavioral Risk Factor Surveillance System 2011–2016, 2018–2019. Madison, WI, United States of America: Wisconsin Department of Health Services.
Wyoming Behavioral Risk Factor Surveillance System	2011–2016, 2018–2019	Centers for Disease Control and Prevention (CDC), Wyoming Department of Health. United States - Wyoming Behavioral Risk Factor Surveillance System 2011–2016, 2018–2019.
United States Behavioral Risk Factor Surveillance System	2011	Centers for Disease Control and Prevention (CDC). United States Behavioral Risk Factor Surveillance System 2011. Atlanta, Georgia: CDC, US Department of Health and Human Services.
United States Behavioral Risk Factor Surveillance System	2012	Centers for Disease Control and Prevention (CDC). United States Behavioral Risk Factor Surveillance System 2012. Atlanta, Georgia: CDC, US Department of Health and Human Services, 2013.
United States Behavioral Risk Factor Surveillance System	2013–2016, 2018–2019	Centers for Disease Control and Prevention (CDC). United States Behavioral Risk Factor Surveillance System 2013–2016, 2018–2019. Atlanta, United States of America: Centers for Disease Control and Prevention (CDC), 2014–2017, 2019–2020.

Title	Years covered	Citation
Gallup Daily	2009–2016	Gallup. United States - Gallup Daily 2009–2016. Washington, D.C., United States of America: Gallup.

Supplementary Table 5. Population data sources

Data source and citation	Description
National Center for Health Statistics, Centers for Disease Control and Prevention, US Census Bureau. United States Bridged-Race Intercensal Population Estimates 2000-2009. Hyattsville, United States: National Center for Health Statistics, Centers for Disease Control and Prevention, 2012. https://www.cdc.gov/nchs/nvss/bridged_race.htm. Accessed October 30, 2012.	Population estimates by county, sex, age, and race and ethnicity for 2000-2009, used in the creation of the post-stratification frame and for aggregating estimates.
National Center for Health Statistics, Centers for Disease Control and Prevention, US Census Bureau. United States Vintage 2020 Bridged-Race Postcensal Population Estimates 2010-2020. Hyattsville, United States: National Center for Health Statistics, Centers for Disease Control and Prevention, 2020. https://www.cdc.gov/nchs/nvss/bridged_race.htm. Accessed February 17, 2022.	Population estimates by county, sex, age, and race and ethnicity for 2010-2020, used in the creation of the post-stratification frame and for aggregating estimates.
Steven Ruggles, Sarah Flood, Matthew Sobek, Danika Brockman, Grace Cooper, Stephanie Richards, and Megan Schouweiler. IPUMS USA: Version 13.0 American Community Survey 1-Year Files, 2009–2021. Minneapolis, MN: IPUMS, 2023. https://doi.org/10.18128/D010.V13.0. Accessed March 8, 2023.	Population by PUMA, sex, age, and educational attainment or marital status for 2007-2019, used in the creation of the post-stratification frame.
Minnesota Population Center. 2000 Census Summary File 3, Table PCT025. IPUMS National Historical Geographic Information System: Version 15.0. Minneapolis, MN: IPUMS 2020. https://www.nhgis.org/. Accessed September 15, 2020.	Population by county, sex, broader age groups (25-34, 35-44, 45-64, 65+), and educational attainment or marital status for 2000, used in the creation of the post-stratification frame.
Steven Ruggles, Sarah Flood, Matthew Sobek, Danika Brockman, Grace Cooper, Stephanie Richards, and Megan Schouweiler. IPUMS USA: Version 13.0 IPUMS Census 5% Sample, 2000. Minneapolis, MN: IPUMS, 2023. https://doi.org/10.18128/D010.V13.0. Accessed August 23, 2022.	Population by PUMA, sex, age, and educational attainment or marital status for 2000, used in the creation of the post-stratification frame.
US Census Bureau. American Community Survey, 2009–2021 American Community Survey 5-Year Estimates, Table B15001; using Census data portal; https://data.census.gov/cedsci/. Accessed April 26, 2023.	Population by county, sex, broader age groups (25-34, 35-44, 45-64, 65+), and educational attainment for 2007-2019, used in the creation of the post-stratification frame.
US Census Bureau. American Community Survey, 2009–2021 American Community Survey 5-Year Estimates, Table B12002; using Census data portal; https://data.census.gov/cedsci/. Accessed April 26, 2023.	Population by county, sex, broader age groups (25-34, 35-44, 45-64, 65+), and marital status for 2007-2019, used in the creation of the post-stratification frame.

Supplementary Table 6. Covariate data sources

Covariate	Data sources	Data processing	Citations
Population density	2000-2019 NCHS bridged race files [1-2]; 2020 cartographic boundary file, state-county for United States [3] accessed using the tigris package [4]	The area of each county was calculated using an Albers Equal Area Conic projection. The total population of each county was divided by the total area of the county and was then log-transformed.	[1] National Center for Health Statistics, Centers for Disease Control and Prevention, US Census Bureau. United States Bridged-Race Intercensal Population Estimates 2000-2009. Hyattsville, United States: National Center for Health Statistics, Centers for Disease Control and Prevention, 2012. https://www.cdc.gov/nchs/nvss/bridged_race.htm. Accessed October 30, 2012.<br>[2] National Center for Health Statistics, Centers for Disease Control and Prevention, US Census Bureau. United States Vintage 2020 Bridged-Race Postcensal Population Estimates 2010-2020. Hyattsville, United States: National Center for Health Statistics, Centers for Disease Control and Prevention, 2020. https://www.cdc.gov/nchs/nvss/bridged_race.htm. Accessed February 17, 2022.<br>[3] US Census Bureau. TIGER/Line Shapefile, 2020 Cartographic Boundary File, State-County for United States, 1:20,000,000. https://www.census.gov/geographies/mapping-files/time-series/geo/tiger-line-file.2020.html#list-tab-790442341. Accessed October 12, 2022.<br>[4] Walker K (2022). *tigris: Load Census TIGER/Line Shapefiles*. R package version 2.0, <https://github.com/walkerke/tigris>.

Covariate	Data sources	Data processing	Citations
Income per capita by race and ethnicity	1990 census [5]; 2000 census [6]; 2009-2021 ACS [7] accessed using the tidycensus package [8]; 2000-2019 Bureau of Labor Statistics, Consumer Price Index [9]	Data were adjusted for inflation using the consumer price index.	[5] Minnesota Population Center. 1990 Census Summary Tape File 4, Table NPB95A. IPUMS National Historical Geographic Information System: Version 15.0. Minneapolis, MN: IPUMS 2020. https://www.nhgis.org/. Accessed August 30, 2020.<br>[6] Minnesota Population Center. 2000 Census Summary File 4, Table NPCT130A. IPUMS National Historical Geographic Information System: Version 15.0. Minneapolis, MN: IPUMS 2020. https://www.nhgis.org/. Accessed August 30, 2020.<br>[7] US Census Bureau. American Community Survey, 2009-2022 American Community Survey 5-Year Estimates, Tables B19013A-B19013I; https://www.census.gov/data/developers/data-sets/acs-5year.html. Accessed April 26, 2023.<br>[8] Walker K, Herman M (2023). *tidycensus: Load US Census Boundary and Attribute Data as 'tidyverse' and 'sf'-Ready Data Frames*. R package version 1.5, <https://walker-data.com/tidycensus/>.<br>[9] US Bureau of Labor Statistics. Consumer Price Index: All Urban Consumers History, All Items 1913-2021. https://www.bls.gov/data/. Accessed October 6, 2022.
Percent of the population age 25 and older who have completed a bachelor's degree by race and ethnicity; Percent of the population age 25 and older who have completed a high school or equivalent degree by race and ethnicity	1990 census [10]; 2000 census [11]; 2010-2021 ACS [12]	ACS estimates for American Indian or Alaskan Native (AIAN), Asian or Pacific Islander (Asian), and Black were not available stratified by Latino ethnicity and were used as proxies for AIAN, Asian, and Black, respectively. Imputation via a small-area estimation model was used to generate and smooth missing values.	[10] Minnesota Population Center. 1990 Census Summary Tape File 4, Table NPB44. IPUMS National Historical Geographic Information System: Version 15.0. Minneapolis, MN: IPUMS 2020. https://www.nhgis.org/. Accessed August 25, 2020.<br>[11] Minnesota Population Center. 2000 Census Summary File 4, Table NPCT064C. IPUMS National Historical Geographic Information System: Version 15.0. Minneapolis, MN: IPUMS 2020. https://www.nhgis.org/. Accessed August 25, 2020.<br>[12] US Census Bureau. American Community Survey, 2010-2021 American Community Survey 5-Year Estimates, Tables C15002A-C15002I; using Census data portal; https://data.census.gov/cedsci/. Accessed April 26, 2023.

Covariate	Data sources	Data processing	Citations
Percent of the population below the poverty line by race and ethnicity	1990 census [13]; 2000 census [14]; 2010-2021 ACS [15]	ACS estimates for American Indian or Alaskan Native (AIAN), Asian or Pacific Islander (Asian), and Black were not available stratified by Latino ethnicity and were used as proxies for AIAN, Asian, and Black, respectively. Imputation via a small-area estimation model was used to generate and smooth missing values.	[13] Minnesota Population Center. 1990 Census Summary Tape File 4, Table NPB100. IPUMS National Historical Geographic Information System: Version 15.0. Minneapolis, MN: IPUMS 2020. https://www.nhgis.org/. Accessed August 30, 2020. [14] Minnesota Population Center. 2000 Census Summary File 4, Table NPCT142A. IPUMS National Historical Geographic Information System: Version 15.0. Minneapolis, MN: IPUMS 2020. https://www.nhgis.org/. Accessed August 30, 2020. [15] US Census Bureau. American Community Survey, 2010-2021 American Community Survey 5-Year Estimates, Tables B17001A-B17001I; using Census data portal; https://data.census.gov/cedsci/. Accessed April 19, 2023.
Percent of the population that was born outside the US by race and ethnicity	1990 census [16]; 2000 census [17]; 2010-2021 ACS [18]	ACS estimates for American Indian or Alaskan Native (AIAN), Asian or Pacific Islander (Asian), and Black were not available stratified by Latino ethnicity and were used as proxies for AIAN, Asian, and Black, respectively. Imputation via a small-area estimation model was used to generate and smooth missing values.	[16] Minnesota Population Center. 1990 Census Summary Tape File 4, Table NPB28. IPUMS National Historical Geographic Information System: Version 15.0. Minneapolis, MN: IPUMS 2020. https://www.nhgis.org/. Accessed October 8, 2020. [17] Minnesota Population Center. 2000 Census Summary File 4, Table NPCT043A. IPUMS National Historical Geographic Information System: Version 15.0. Minneapolis, MN: IPUMS 2020. https://www.nhgis.org/. Accessed October 8, 2020. [18] US Census Bureau. American Community Survey, 2010-2021 American Community Survey 5-Year Estimates, Tables B05003A-B05003I; using Census data portal; https://data.census.gov/cedsci/. Accessed April 26, 2023.

Supplementary Table 7. Modelled causes of morbidity

Cause	Covariates	Prop. Total YLDs[1]
Communicable, maternal, neonatal, and nutritional diseases	Standard set[2] + cause-specific YLL rate	0.037
Neoplasms	Standard set + cause-specific YLL rate	0.021
Cardiovascular diseases	Standard set + cause-specific YLL rate	0.042
Chronic respiratory diseases	Standard set + asthma + COPD + cause-specific YLL rate	0.051
Digestive diseases	Standard set + cause-specific YLL rate	0.017
Neurological disorders	Standard set + cause-specific YLL rate	0.076
Mental disorders	Standard set + cognitive difficulty + depression + stress + worry	0.157
Substance use disorders	Standard set + depression + stress + worry + cause-specific YLL rate	0.072
Diabetes and kidney diseases	Standard set + diabetes + cause-specific YLL rate	0.075
Skin and subcutaneous diseases	Standard set + cause-specific YLL rate	0.040
Sense organ diseases	Standard set + vision or hearing disability	0.059
Musculoskeletal disorders	Standard set + arthritis + cause-specific YLL rate	0.228
Other non-communicable diseases	Standard set + severe tooth loss + cause-specific YLL rate	0.055
Transport injuries	Standard set + cause-specific YLL rate	0.016
Unintentional injuries	Standard set + cause-specific YLL rate	0.050
Self-harm and interpersonal violence	Standard set + cause-specific YLL rate	0.004
		Total: 1.000

[1]National, all ages and sexes, 2019; GBD 2021
[2]Standard covariate set included: frequent activity limitation, pain, personal care difficulty, physical difficulty, population density, income per capita, educational attainment (high school or higher, bachelor's degree or higher), poverty, and born outside the US.

Supplementary Table 8. Population mask

	AIAN			Asian			Black			Latino			White			Total		
Stratum	Counties masked	Person-years masked	Percent person-years in stratum	Counties masked	Person-years masked	Percent person-years in stratum	Counties masked	Person-years masked	Percent person-years in stratum	Counties masked	Person-years masked	Percent person-years in stratum	Counties masked	Person-years masked	Percent person-years in stratum	Counties masked	Person-years masked	Percent person-years in stratum
Total	2636 (84.8%)	9.1* (17.8%)		2443 (78.6%)	8.5* (2.6%)		1622 (52.2%)	7.5* (1.0%)		1632 (52.5%)	11.4* (1.2%)		59 (1.9%)	0.8* (0.0%)		31 (1.0%)	0.4* (0.0%)	
Urban or rural code																		
Large central metro	8 (10.1%)	0.1 (0.7%)	16.5%	0 (0.0%)	0.0 (0.0%)	51.8%	0 (0.0%)	0.0 (0.0%)	42.1%	0 (0.0%)	0.0 (0.0%)	49.5%	0 (0.0%)	0.0 (0.0%)	22.1%	0 (0.0%)	0.0 (0.0%)	30.6%
Large fringe metro	309 (78.0%)	1.5 (28.7%)	10.4%	184 (46.5%)	0.9 (1.1%)	24.7%	83 (21.0%)	0.5 (0.3%)	20.8%	103 (26.0%)	0.7 (0.4%)	17.2%	0 (0.0%)	0.0 (0.0%)	24.8%	0 (0.0%)	0.0 (0.0%)	23.0%
Medium metro	359 (73.6%)	1.5 (14.9%)	19.5%	239 (49.0%)	0.9 (1.7%)	15.9%	115 (23.6%)	0.5 (0.4%)	19.0%	121 (24.8%)	0.7 (0.4%)	19.8%	1 (0.2%)	0.0 (0.0%)	22.1%	0 (0.0%)	0.0 (0.0%)	21.0%
Small metro	356 (80.9%)	1.3 (22.0%)	11.6%	241 (54.8%)	1.0 (8.1%)	3.8%	125 (28.4%)	0.5 (0.9%)	6.9%	149 (33.9%)	0.8 (1.4%)	5.9%	2 (0.5%)	0.0 (0.0%)	10.4%	1 (0.2%)	0.0 (0.0%)	8.9%
Micropolitan	717 (87.5%)	2.1 (21.4%)	19.5%	693 (84.6%)	3.5 (37.0%)	2.9%	384 (46.9%)	2.4 (4.9%)	6.1%	307 (37.5%)	2.3 (4.8%)	4.9%	9 (1.1%)	0.1 (0.0%)	11.2%	6 (0.7%)	0.0 (0.0%)	9.2%
Noncore	1694 (92.7%)	2.6 (22.5%)	22.4%	1805 (98.8%)	2.2 (83.2%)	0.8%	1340 (73.3%)	3.6 (9.1%)	5.1%	1399 (76.6%)	6.8 (25.7%)	2.7%	59 (3.2%)	0.7 (0.2%)	9.4%	31 (1.7%)	0.4 (0.1%)	7.4%
Census region																		
South	1246 (88.6%)	3.9 (23.5%)	32.0%	1137 (80.8%)	4.0 (5.7%)	21.6%	451 (32.1%)	2.8 (0.6%)	56.1%	686 (48.8%)	5.4 (1.5%)	35.9%	19 (1.4%)	0.2 (0.0%)	34.8%	8 (0.6%)	0.1 (0.0%)	37.0%
West	269 (62.4%)	1.3 (5.6%)	44.3%	309 (71.7%)	1.2 (0.8%)	46.5%	319 (74.0%)	1.2 (1.6%)	9.2%	152 (35.3%)	0.9 (0.2%)	40.7%	18 (4.2%)	0.3 (0.0%)	19.5%	9 (2.1%)	0.1 (0.0%)	23.2%
Northeast	172 (79.3%)	0.9 (30.6%)	6.0%	110 (50.7%)	0.8 (1.3%)	20.0%	69 (31.8%)	0.6 (0.4%)	16.4%	67 (30.9%)	0.7 (0.5%)	14.1%	0 (0.0%)	0.0 (0.0%)	19.3%	0 (0.0%)	0.0 (0.0%)	18.0%
Midwest	949 (90.0%)	3.0 (33.5%)	17.7%	887 (84.1%)	2.5 (6.5%)	11.9%	783 (74.2%)	3.0 (2.1%)	18.4%	727 (68.9%)	4.4 (4.9%)	9.2%	22 (2.1%)	0.3 (0.0%)	26.4%	14 (1.3%)	0.2 (0.0%)	21.8%

*Person-years are given in millions.

Modelled HALE estimates were masked (not displayed) in all years for county and racial and ethnic populations with a mean annual population of fewer than 1000 people from 2000 to 2019. The number of unique counties masked (and corresponding percentage of all counties), the person-years in millions represented by these county-years (and corresponding percentage of all person-years), and the percentage of the person-years masked in each stratum are listed in this table. AIAN = American Indian or Alaska Native.

Supplementary Figures

Supplementary Figure 1. Data and modelling flowchart.

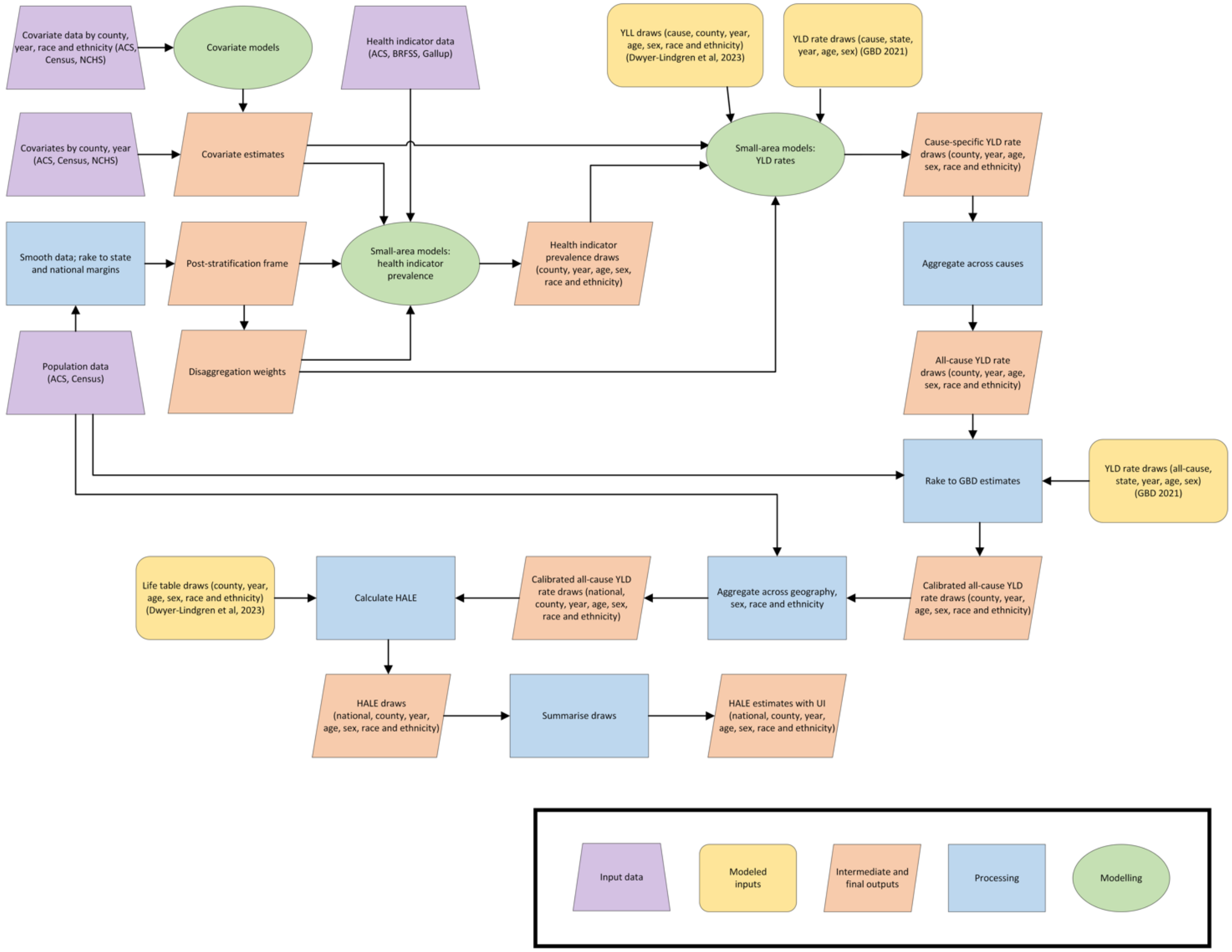

HALE: Healthy life expectancy; YLL: years of life lost; YLD: years lived with disability; ACS: American Community Survey; NCHS: National Center for Health Statistics; BRFSS: Behavioral Risk Factor Surveillance System; GBD: Global Burden of Disease study; UI: uncertainty interval.

Supplementary Figure 2. Hyperprior sensitivity analysis (YLD indicators).

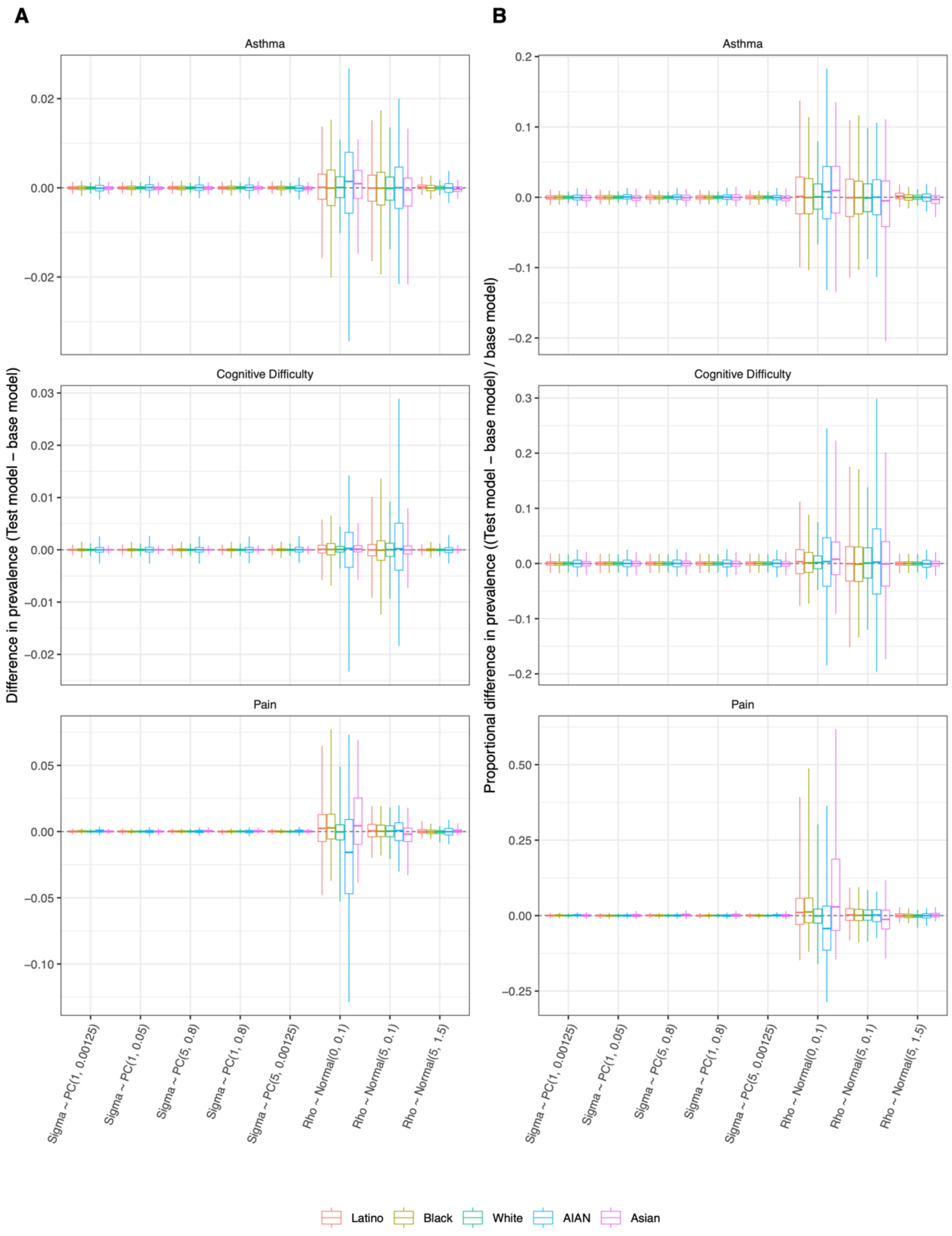

Comparisons are for county-level estimates among counties with unmasked estimates, by age, sex, and year, relative to the base model described in the Supplementary Methods, section 4.1. Boxes indicate the 1st, 25th, 50th, 75th, and 99th percentiles of the differences. Note that y-axis scales differ among indicators.

Supplementary Figure 3. Hyperprior sensitivity analysis (YLD models).

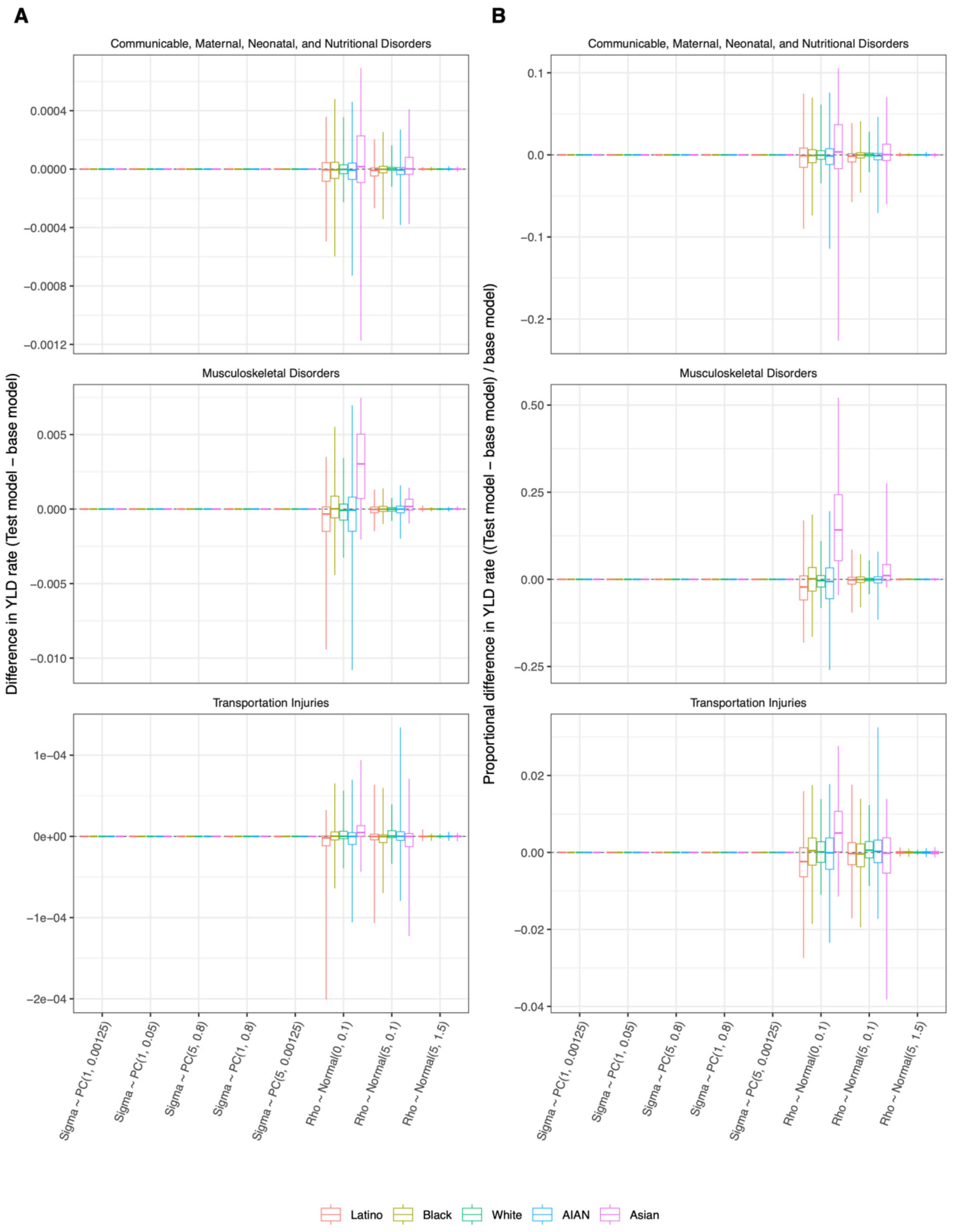

Comparisons are for county-level estimates among counties with unmasked estimates, by age, sex, and year, relative to the base model described in the Supplementary Methods, section 5.3. Boxes indicate the 1st, 25th, 50th, 75th, and 99th percentiles of the differences. Note that y-axis scales differ among indicators.
